# In vitro proliferation and long-term preservation of functional primary rat hepatocytes in cell fibers

**DOI:** 10.1038/s41598-022-12679-3

**Published:** 2022-05-25

**Authors:** Elsa Mazari-Arrighi, Teru Okitsu, Hiroki Teramae, Hoshimi Aoyagi, Mahiro Kiyosawa, Mariko Yano, François Chatelain, Alexandra Fuchs, Shoji Takeuchi

**Affiliations:** 1 Institute of Industrial Science, The University of Tokyo, 4-6-1 Komaba, Meguro-Ku, Tokyo, 153-8505 Japan; 2 ERATO Takeuchi Biohybrid Innovation Project, Japan Science and Technology Agency (JST), Saitama, Japan; 3 LIMMS/CNRS-IIS UMI 2820, Institute of Industrial Science, The University of Tokyo, Tokyo, Japan; 4 Faculty of Teacher Education, Shumei University, Chiba, Japan; 5 Université de Paris, Inserm, U976 HIPI, 75006 Paris, France; 6 AP-HP, Hôpital Saint-Louis, Paris, France; 7 IRIG/DRF, CEA, 38000 Grenoble, France

**Keywords:** Biomedical engineering, Tissue engineering, Biophysical methods

## Abstract

Primary hepatocytes are essential cellular resources for drug screening and medical transplantation. While culture systems have already succeeded in reconstituting the biomimetic microenvironment of primary hepatocytes, acquiring additional capabilities to handle them easily as well as to expand them remains unmet needs. This paper describes a culture system for primary rat hepatocytes, based on cell fiber technology, that brings scalability and handleability. Cell fibers are cell-laden core–shell hydrogel microfibers; in the core regions, cells are embedded in extracellular matrix proteins, cultured three-dimensionally, and exposed to soluble growth factors in the culture medium via the hydrogel shells. By encapsulating primary rat hepatocytes within cell fibers, we first demonstrated their proliferation while maintaining their viability and their hepatic specific functions for up to thirty days of subsequent culture. We then demonstrated the efficiency of proliferating primary rat hepatocytes in cell fibers not only as cell-based sensors to detect drugs that damage hepatic functions and hepatocellular processes but also as transplants to improve the plasma albumin concentrations of congenital analbuminemia. Our culture system could therefore be included in innovative strategies and promising developments in applying primary hepatocytes to both pharmaceutical and medical fields.

## Introduction

Primary hepatocytes are currently used for fundamental studies as well as for pharmaceutical and medical applications including drug screening and hepatocyte transplantation^1–3^. Since the quality of hepatocytes is crucial for these applications, over the past few years, culture systems for primary hepatocytes have made progress not only in long-term maintenance of their viability and their metabolic functions but also in allowing them to proliferate. In fact, these systems tend to enable primary hepatocytes in vitro to behave similarly to how they do in vivo: in the liver, functional hepatocytes retain their viability with a lifespan of over three months^4^, while they proliferate by dividing until reaching the adequate cellular volume necessary for the liver to be restored when liver suffers from any change of losing its volume by injury, infection, or surgical procedure of partial hepatectomy^1^. Therefore, culture systems for primary hepatocytes have been developed to reproduce the microenvironment of hepatocytes in the liver, and three key factors have been identified: (i) three-dimensional culture^5,6^, (ii) extracellular matrix (ECM) proteins^7,8^, and (iii) soluble growth factors^9–11^. Recently, Rose et al. reported successful in vitro proliferation of primary human hepatocytes while maintaining their metabolic functions for four weeks by embedding them in an ECM of collagen in which hepatocytes formed spheroid-shaped clusters when exposed to hepatocyte growth factor (HGF), epidermal growth factor (EGF), and insulin-transferrin selenium (ITS) in the medium^12^.

Once the culture system for primary hepatocytes has reconstituted this biomimetic microenvironment, the next key progress would be to acquire the capabilities of both scalability and handleability for implementation into pharmaceutical and medical fields, as drug screening and hepatocyte transplantation require not only large numbers of hepatocytes^13–17^ but also easy-to-handle and transferable constructs in which hepatocytes are cultured^17,18^. However, very few investigations have been performed to yield such a scalable and handleable culture system for primary hepatocytes; although core–shell hydrogel microcapsules might be good strategy^19–21^, they still lack the evidence to show the potential of their practical use through providing primary hepatocytes with a biomimetic culture environment accompanied with both scalability and handleability.

Cell fiber technology is a useful system to culture cells three-dimensionally for long periods; in cell fibers, cells can proliferate, migrate, and connect with each other to form functional cellular tissue^22^. Cell fibers are cell-laden core–shell hydrogel microfibers and they are generated by using a double-coaxial laminar-flow microfluidic device; the core contains both cells and ECM proteins while the shell consists of an alginate hydrogel. All the cells in the core can easily access oxygen and nutrients in the culture medium since the diameter of the cell fiber is kept at a few hundreds of micrometres over its entire length. Cell fibers have been shown to allow three-dimensional tissue formation of various types of cells including cardiomyocytes, vascular endothelial cells, nerve cells, smooth muscle cells and adipocytes^22–25^. The advantage of cell fiber technology is to be mass-producible and scalable; a cell fiber of considerable length (meter-range) can be fabricated in a short time (a few minutes) and cell fibers have allowed pluripotent stem cells to increase in number while keeping their original characteristics^24^. The other advantage of cell fibers is to be handleable; cell fibers encapsulating pancreatic islet cells have been stably and reproducibly transplanted to diabetic mice and even retrieved from these animals^22^.

For this study, we hypothesized that cell fibers could be used to develop a culture system for primary hepatocytes that is equipped with scalability and handleability, in addition to the already available culture biomimetic environment that allows primary hepatocytes both to proliferate and to maintain their viability and their metabolic functions. We first describe our encapsulation technique of primary rat hepatocytes into core–shell hydrogel microfibers to form cell fibers. We pursued by evaluating whether primary rat hepatocytes cultured in cell fibers can proliferate and can subsequently preserve their viability and their functionality for up to 30 days. We then demonstrate pharmaceutical relevance of the primary rat hepatocytes cultured in cell fibers for screening drugs that might damage hepatic functions and hepatocellular processes such as proliferation. Finally, to show scalability and handleability of our culture system for the treatment of the diseases due to hepatocellular malfunction, we assess the efficiency of primary rat hepatocytes cultured in cell fibers as transplants to improve the plasma albumin concentrations in analbumenic rats.

## Results

### Applying cell fiber technology to encapsulate primary rat hepatocytes within cell fibers

**Figure 1:**
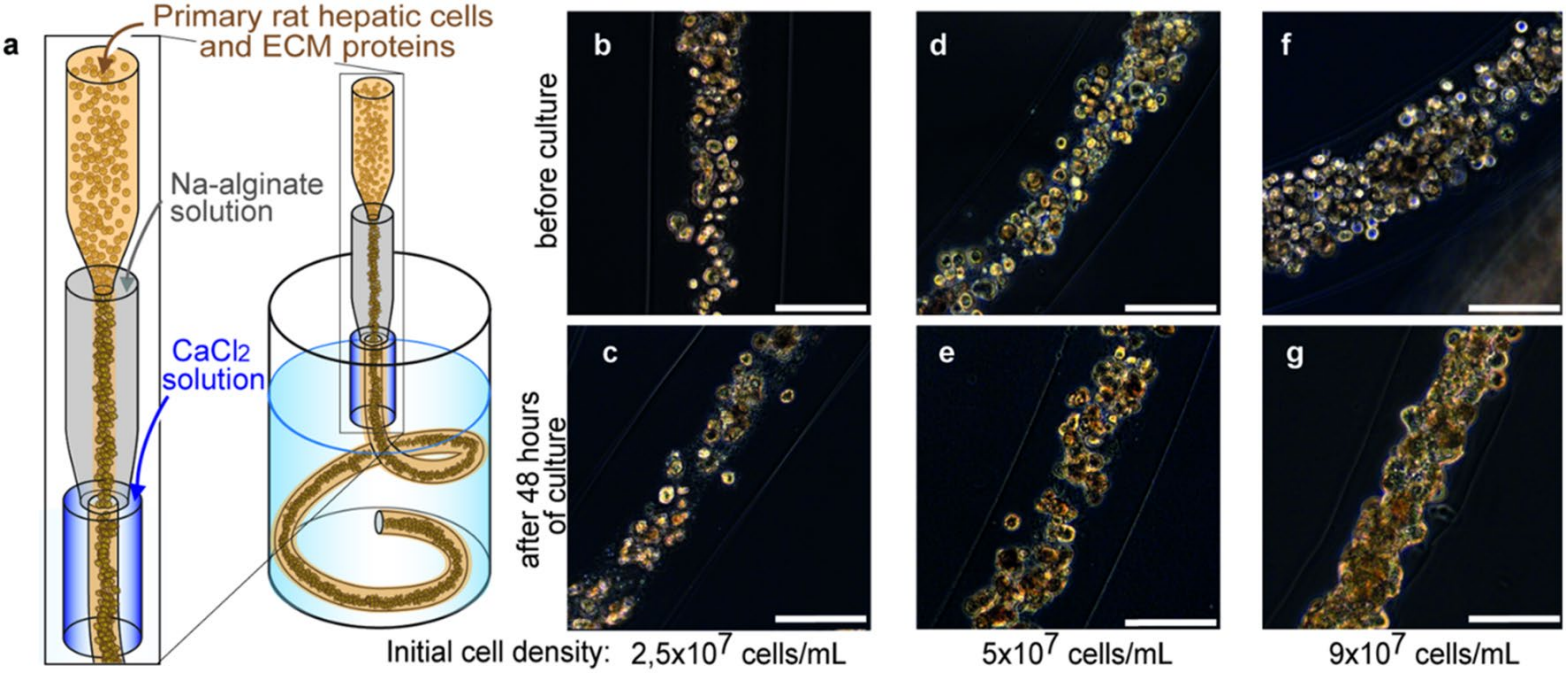
Encapsulating primary rat hepatocytes within core–shell hydrogel microfibers by applying cell fiber technology. (**a**) Schematic drawing of the fabrication of core–shell hydrogel microfibers encapsulating freshly isolated rat hepatocytes through a double co-axial microfluidic device. (**b–g**) Representative dark-field images (n = 12 cell fibers for each group) of primary rat hepatocytes encapsulated in cell fibers before culture and after 48 h of culture in three experimental groups possessing different initial cell seeding densities: 2.5 × 10^7^ cells mL^−1^, 5 × 10^7^ cells mL^−1^, and 9 × 10^7^ cells mL^−1^. Scale bars; 100 µm.

To embed primary rat hepatocytes in ECM proteins acting as a three-dimensional culture environment, we used our previously developed cell fiber technology^22^. First, we evaluated the impact of the initial cell seeding density of primary rat hepatocytes both on how they would occupy the space of the core region of cell fibers and on how they would behave there afterwards. For this evaluation, core–shell hydrogel microfibers were fabricated to encapsulate primary rat hepatocytes through a double-coaxial laminar-flow microfluidic device (Fig. 1a) by using ECM conditions that we optimized beforehand [a mixture of type I collagen (Native collagen) and Matrigel (Fig. S1)] and by using the following three initial cell seeding densities of primary rat hepatocytes: 2.5 × 10^7^, 5 × 10^7^, and 9 × 10^7^ cells mL^-1^. Through microscopic observations just after cell fiber fabrication, we found that, when the initial cell seeding density was 9 × 10^7^ cells mL^−1^, the encapsulated cells nearly fully occupied the core regions of the cell fibers, and the space of the core regions occupied by the cells decreased as expected as a function of the initial cell seeding density (Fig. 1b,d,f). After 48 h of culture without any stimulation of proliferation, we found in their core regions that the encapsulated cells self-assembled into different cellular clusters in terms of shape and size, depending on each initial cell seeding density; at 9 × 10^7^ cells mL^−1^, primary rat hepatocytes formed fiber-shaped clusters; at 5 × 10^7^ cells mL^−1^, they formed rod-shaped clusters; at 2.5 × 10^7^ cells mL^−1^, they formed mostly spheroid-shaped clusters (Fig. 1c,e,g). These results indicate that primary rat hepatocytes cultured in cell fibers cluster spontaneously during the first 48 h after being encapsulated, and that the shape as well as the size of resulting cellular clusters would vary based on the initial cell density embedded in the core region: within a certain window of the initial cell density, the higher the density, the more likely cellular clusters would take a fiber shape. In contrast, the lower is the cell density, the more likely they would be spheroid-shaped.

### In vitro proliferation and long-term maintenance of functional primary rat hepatocytes in cell fibers

We hypothesized that cell fiber could create an appropriate microenvironment for soluble factor stimulation to trigger the proliferation of encapsulated primary rat hepatocytes. Before testing this hypothesis, as described in the literature^9–12^, we prepared the previously identified soluble factors including HGF and EGF to stimulate the proliferation. Eventually, we decided to use conditioned medium collected from 3T3 cells (3T3CM) that had been cultured in cell fibers for 5 days, because we confirmed that the amount of HGF measured in the 3T3CM is the highest after 5 days of culture (Fig. S2g), and also that this 3T3CM is comparable with the medium supplemented with not only recombinant mouse HGF but also recombinant mouse EGF (Fig. S3m,n).

**Figure 2:**
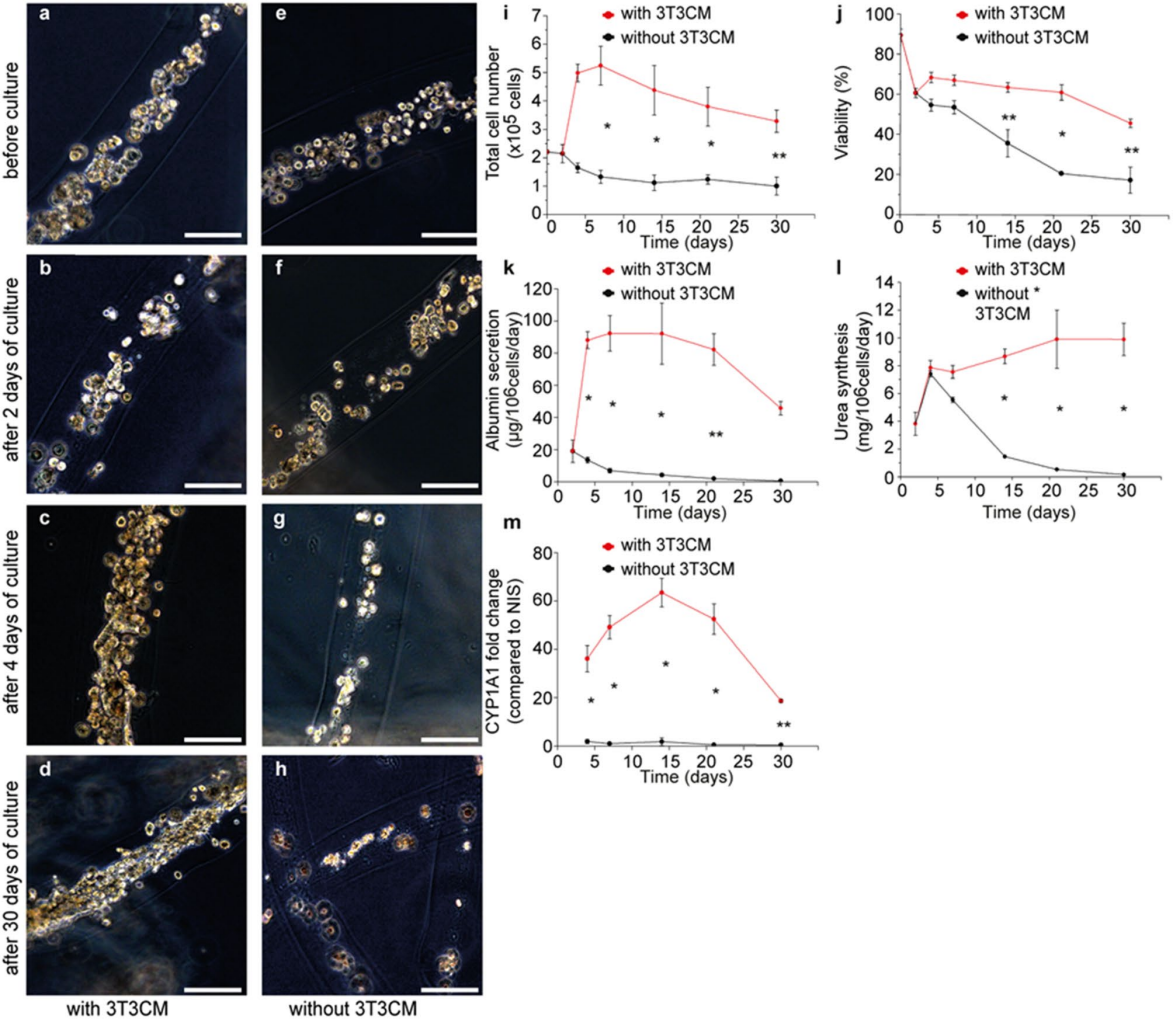
Culture of primary rat hepatocytes in cell fibers for up to 30 days either with or without using 3T3CM. (**a**–**h**) Representative dark-field images of primary rat hepatocytes encapsulated in cell fibers (n = 12 for each group) at the initial cell seeding density of 2.5 × 10^7^ cells mL^−1^ before culture, after 2 days, after 4 days, and 30 days of culture in two experimental groups: (**a**–**d**) in one group 3T3CM was used after 2 days of culture, (**e**–**h**) while in the other group 3T3CM was not used. Scale bars; 100 µm. (**i**–**m**) Time course of the characteristics including hepatocyte-specific functions of primary rat hepatocytes cultured in cell fibers (n = 4 per data point) for over 30 days of culture either with or without using 3T3 CM: (**i**) total cell number, (**j**) viability, (**k**) albumin secretion, (**l**) urea synthesis, (**m**) CYP1A1 enzyme activity. Error bars denote mean ± SD. *P < 0.01 and **P < 0.05; Student’s t-test at each time point.

Then, to test our hypothesis, primary rat hepatocytes were encapsulated in core–shell hydrogel microfibers at the initial cell seeding density of 2.5 × 10^7^ cells mL^−1^. This value was determined based on the findings of our previous experiments showing that such initial cell seeding density secures space for the cultured primary rat hepatocytes to increase in number within cell fibers (Fig. 1b,d). After 2 days of culture, cell fibers encapsulating primary rat hepatocytes at 2.5 × 10^7^ cells mL^−1^ were randomly divided into two groups: (1) in the experimental group, cell fibers started to be cultured in media containing 50% of 3T3CM, (2) in the control group, cell fibers were cultured in the same medium as the one used for the first 2 days of culture. Subsequently, we assessed the morphologies of primary rat hepatocytes cultured in these cell fibers, by measuring various characteristics including hepatocyte-specific functions: (i) cell number, (ii) viability, (iii) albumin secretion, (iv) urea synthesis, (v) CYP1A1 enzyme activity, and we compared these characteristics between the experimental and the control groups for up to 30 days of culture. Regarding microscopic morphologies, as expected, primary rat hepatocytes cultured in cell fibers for up to 2 days assemble spontaneously into spheroid-shaped clusters that were scattered in the core regions of cell fibers (Fig. 2b,f). After 4 days of culture, we observed that primary rat hepatocytes occupied almost the entire space of the core regions and formed fiber-shaped aggregates in the experimental group, while they remained as sparsely scattered spheroid-shaped clusters in the control group (Fig. 2c,g). Furthermore, we found in the experimental group that the cell number increased 2.4 ± 0.7 times higher after 4 days of culture than after 2 days of culture and that the viability of 45.8 ± 2.0% was maintained up to 30 days of culture. In contrast, in the control group neither did the cell number increase, nor was the viability maintained (Fig. 2i,j). We also found that primary rat hepatocytes maintained their hepatocyte-specific functions (albumin secretion, urea synthesis, CYP1A1 enzyme activity) up to 30 days in culture in the experimental group, but not in the control group (Fig. 2k–m).

**Figure 3:**
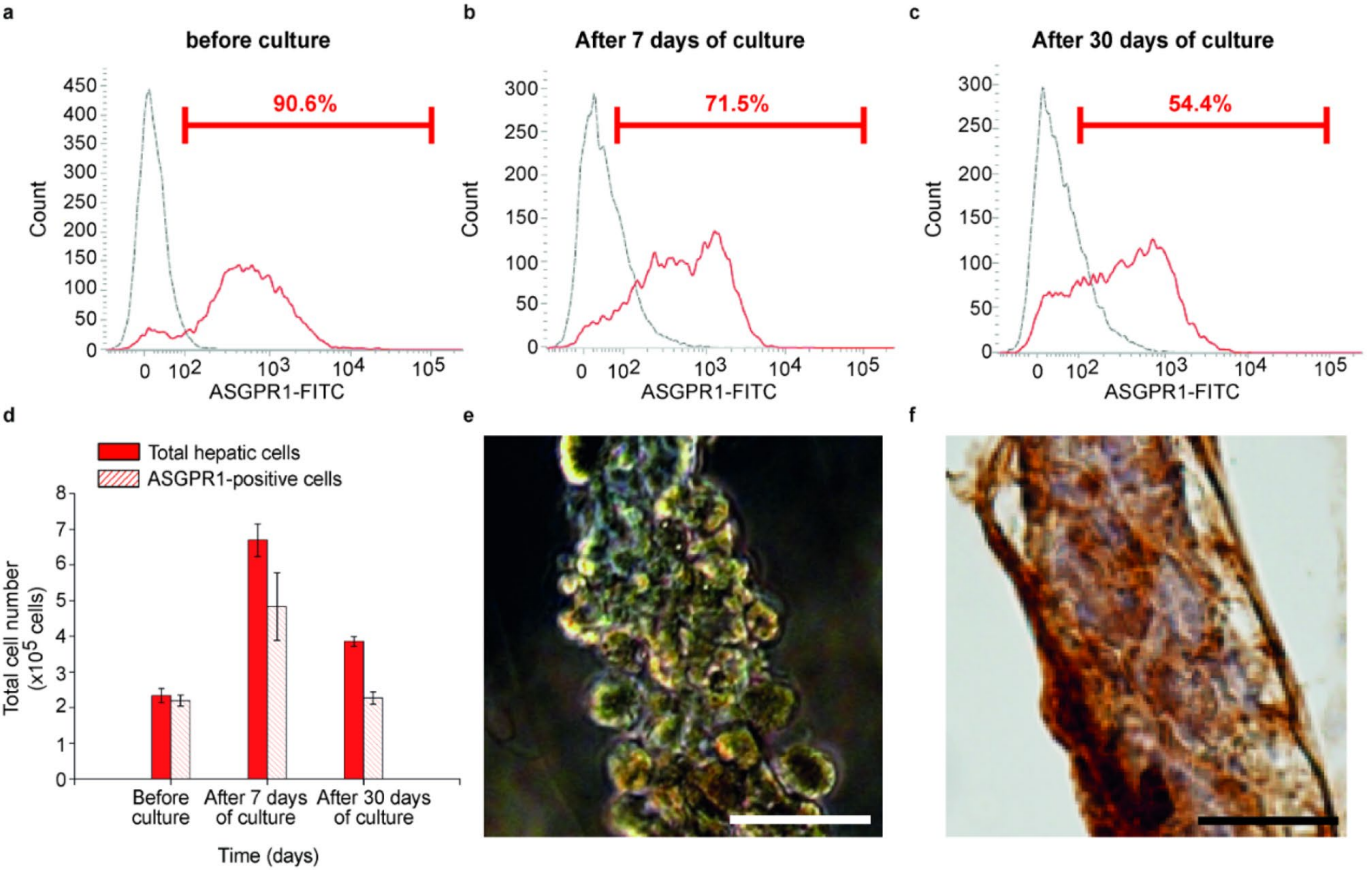
Hepatocyte detection in primary rat hepatic cells cultured in cell fibers using 3T3CM. (**a**–**c**) Representative FACS histograms (of 3 samples at least for each culture duration) showing the surface marker of asialoglycoprotein receptor-1 (ASGPR-1) on the primary rat hepatocytes (**a**) before culture, (**b**) after 7 days, (**c**) and 30 days of culture in cell fibers using 3T3CM. Red lines represent samples that were treated using both of a mouse-anti-human ASGPR1 antibody and a FITC-labelled goat anti-mouse antibody, while grey lines represent samples that were treated only with a FITC-labelled goat anti-mouse antibody. Red numbers in FACS histograms represent the percentage of cells that are positive for ASGPR-1. (**d**) Total cell number and ASGPR-1 positive cell number in primary rat hepatocytes before culture, after 7 days, and 30 days of culture (n = 3 at least per each time point). (**e**) Representative dark-field image (n = 3) and (**f**) representative image of immunohistochemical analysis for albumin localization (n = 3) of primary rat hepatocytes that were cultured for 7 days in cell fibers using 3T3CM. Scale bars; 50 µm.

Thereafter, we confirmed whether the cells in the experimental group, which had proliferated under stimulation in cell fibers, were hepatocytes or not. We analyzed the expression of a specific marker of hepatocyte, ASGPR-1, on the encapsulated cells in cell fibers using flow cytometry^26^ and we also attempted to detect albumin in those cells by immunohistochemistry. We measured that the number of ASGPR-1 expressing cells were 219% higher after 7 days of culture than before stimulation, and that the number of ASGPR-1 expressing cells were still 104% higher after 30 days of culture than before stimulation (Fig. 3a–d, Table S1). We also found that almost all encapsulated cells that had been cultured in the cell fibers for 7 days were albumin positive and seemed to connect with each other (Fig. 3e,f).

Taken together, these results clearly show that primary rat hepatocytes can survive for long periods while maintaining their proper functions in cell fibers only when they totally fill the core space by proliferating during the first 4 days of culture. These results also indicate that primary rat hepatocytes need to connect with each other and form cellular aggregates in order to acquire a three-dimensional culture environment, as interaction of primary rat hepatocytes with ECM proteins alone is not sufficient to allow them to survive in cell fibers for long periods.

### In vitro detection of drug hepatotoxicity using primary rat hepatocytes in cell fibers

To explore the possible application of cell fibers encapsulating primary hepatocytes in the field of drug screening, we evaluated the potential of cell fibers for in vitro detection of hepatotoxicity of drugs. For this evaluation, we attempted to obtain the respective concentrations of a 50% inhibitory effect (IC50 values) for two kinds of well-known hepatotoxic compounds: acetaminophen and diclofenac. First, primary rat hepatocytes were encapsulated into core–shell hydrogel microfibers at the initial cell seeding density of 2.5 × 10^7^ cells mL^−1^ and were cultured in the media supplemented with 3T3CM. Secondly, the concentration-reaction curves for these compounds were obtained through the assays of cellular viability, albumin secretion, and urea synthesis performed at different time points: 4 days, 7 days, 14 days, and 30 days after the start of culture. Thirdly, based upon these concentration-reaction curves (Figs. S4–S7), the corresponding IC50 values were estimated. Moreover, we attempted to compare these IC50 values with those obtained using another culture system where primary rat hepatocytes were cultured on collagen-coated 24-well plates. We found that the IC50 values for both acetaminophen and diclofenac can be estimated up to 30 days of culture when using cell fibers, and that these estimated IC50 values for each compound are both reproducible in individual cell fibers as well as stable over time regardless of these three assays, while the IC50 values for either of the two compounds become unable to be estimated after 7 days of culture when using collagen-coated 24-well plates (Table 1). We also found that the IC50 values for both the compounds obtained using cell fibers are in good accordance with the IC50 values reported previously in the literature (Tables S2–S3)^27–30^. These findings indicate that the culture system using cell fibers encapsulating primary rat hepatocytes could be useful to estimate drug hepatotoxicity in the field of drug screening and might even improve the reliability of the estimation because of the reproducibility and the chronological stability of the primary rat hepatocytes cultured in the cell fibers (Table S4).

**Table 1:** 50% inhibitory effect (IC50 values) for acetaminophen and diclofenac relating to viability, albumin secretion, and urea synthesis of primary rat hepatocytes cultured either in cell fibers for 4 days, 7 days, 14 days, and 30 days, or in collagen-coated 24-well plates for 4 days and 7 days.

Compound	Culture system	Culture duration (days)	IC50 value (µM)		
			Assay methods		
			Viability	Albumin secretion	Urea synthesis
Acetaminophen	Core–shell hydrogel microfibres	4	18,324 ± 509	16,703 ± 2540	17,034 ± 1045
		7	18,556 ± 661	16,406 ± 992	15,886 ± 582
		14	18,919 ± 772	16,273 ± 1289	15,943 ± 490
		30	18,853 ± 661	15,876 ± 2063	17,299 ± 853
	Collagen-coated 24-well plates	4	19,277 ± 164	8507 ± 89	24,038 ± 57
		7	N/A	N/A	N/A
Diclofenac	Core–shell hydrogel microfibres	4	332 ± 17	282 ± 9	350 ± 12
		7	312 ± 21	310 ± 3	315 ± 9
		14	339 ± 12	299 ± 5	322 ± 7
		30	345 ± 25	337 ± 13	336 ± 16
	Collagen-coated 24-well plates	4	472 ± 98	N/A	385 ± 41
		7	N/A	N/A	N/A

Data are presented as the mean ± standard deviation of at least four independent cell fibers and four collagen-coated wells from collagen-coated 24-well plates.

### In vitro detection of drug inhibition of hepatic regeneration using primary rat hepatocytes in cell fibers

**Figure 4:**
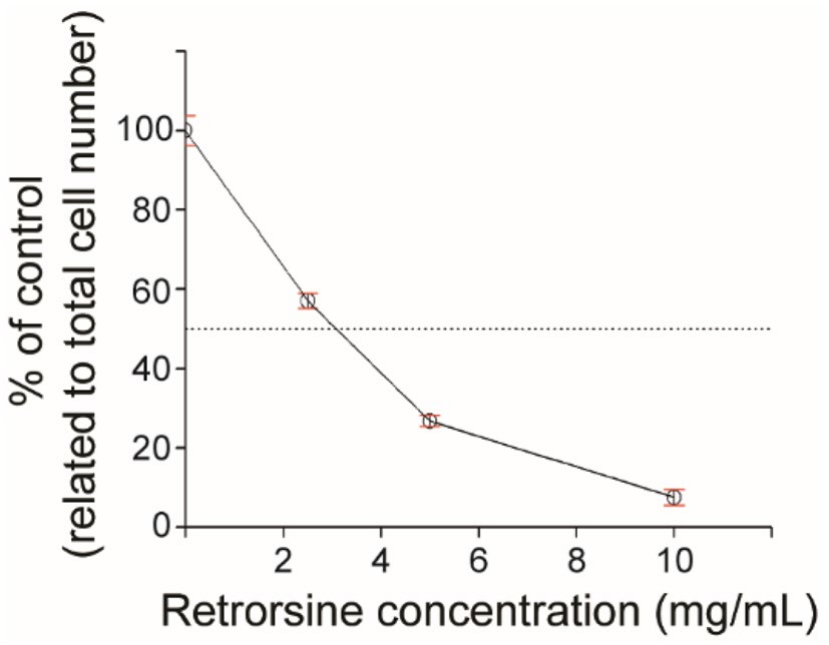
Retrorsine concentration-reaction curve relating to inhibition of primary rat hepatocyte proliferation in cell fibers in vitro. The effect of 24-h retrorsine treatment on inhibition of primary rat hepatocyte proliferation in cell fibers was evaluated using three different concentrations (2.5 mg mL^−1^, 5 mg mL^−1^, 10 mg mL^−1^) of retrorsine (n = 5 per data point). Error bars denote mean ± SD.

Since we have demonstrated that primary rat hepatocytes can proliferate in cell fibers while keeping their hepatocyte-specific functions, we hypothesized that these cell fibers could serve for screening drugs that inhibit hepatic regeneration. To test this hypothesis, we used retrorsine, a compound that is standardly used to inhibit rat hepatocyte regeneration in vivo^31,32^ and we attempted to measure its IC50 value by obtaining its concentration-reaction curve. Practically, core–shell hydrogel microfibers were fabricated to encapsulate primary rat hepatocytes at the initial cell seeding density of 2.5 × 10^7^ cells mL^−1^. Then, the fabricated cell fibers were randomly divided into four groups; (1) in the control group, the cell fibers were cultured in media containing 3T3CM after 2 days of culture, (2) in the three experimental groups, the cell fibers were cultured under similar conditions as the control group except that they were exposed to three different concentrations of retrorsine (2.5 mg mL^−1^, 5 mg mL^−1^, 10 mg mL^−1^) for 24 h after 2 days of culture. The proliferation ratio of primary hepatocytes in the experimental groups were calculated, and the concentration-reaction curve for retrorsine was obtained (Fig. 4). Using the curve, we found that the IC50 value for retrorsine is estimated to be 3.1 ± 0.1 mg mL^−1^ (~ 8.82 ± 0.3 mM).

**Figure 5:**
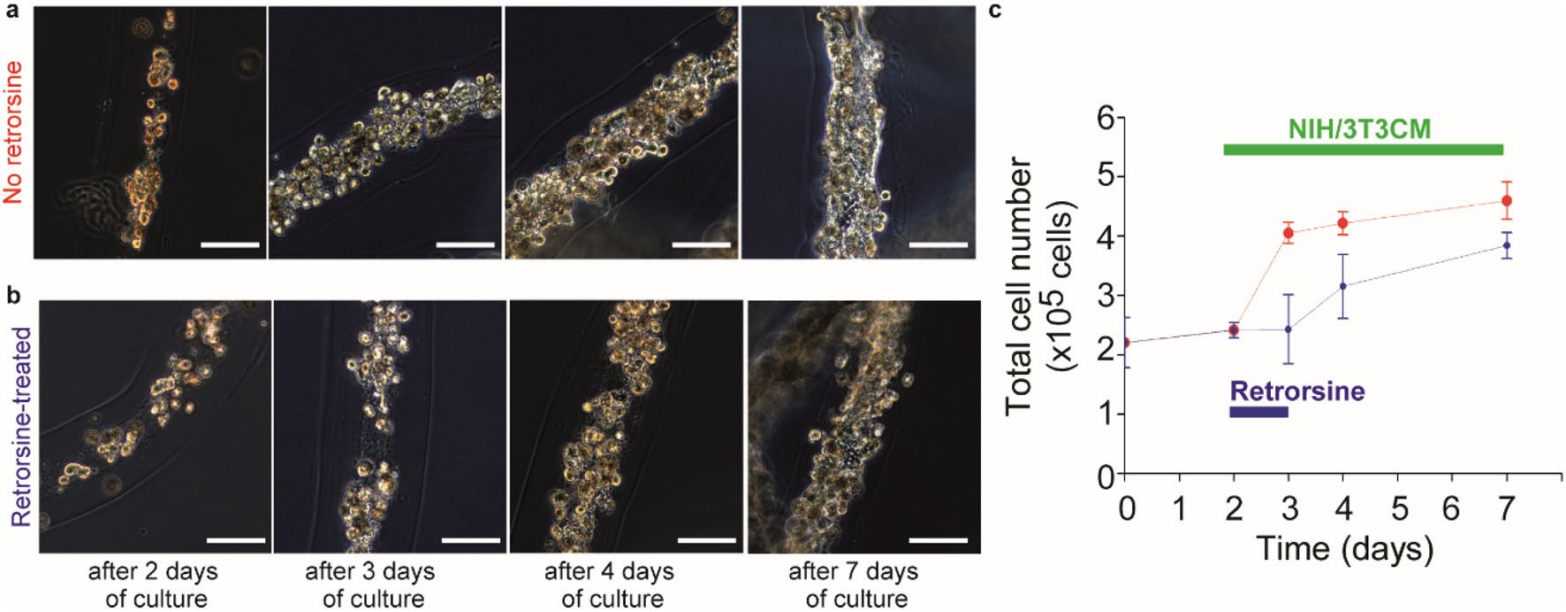
Proliferation of primary rat hepatocytes after 24-h treatment of retrorsine in cell fibers. (**a**, **b**) Representative dark-field images of primary rat hepatocytes cultured in cell fibers (n = 5 for each group) for 2 days, 3 days, 4 days, and 7 days using 3T3CM. Cell fibers encapsulating primary hepatocytes were divided into two groups: (**a**) in one group the hepatocytes were not treated with retrorsine, (**b**) while in the other group they were treated with retrorsine for 24-h after 2 days of culture. Scale bars; 100 µm. (**c**) Time course of total cell number of primary rat hepatocytes cultured in cell fibers using 3T3CM either (**c**) with or (**d**) without 24-h retrorsine treatment after 2 days of culture (n = 5 per data point). Error bars denote mean ± SD.

Subsequently, we evaluated whether this inhibitory effect of retrorsine on hepatocyte proliferation is reversible or irreversible. For that, we first fabricated cell fibers encapsulating primary rat hepatocytes at the initial cell seeding density of 2.5 × 10^7^ cells mL^−1^. Then, after 2 days of culture, we randomly divided them into two groups; in the control group, the cell fibers were cultured in media containing 3T3CM for up to 5 days of additional culture; in the experimental group, the cell fibers were cultured under similar conditions as the control group except that they were exposed to retrorsine at a concentration of 10 mg mL^−1^ during 24 h from day 2 of culture to day 3 of culture. Subsequently, we compared these two groups over time by analyzing their morphology and cell number. We found that primary rat hepatocytes in the experimental group proliferated after the removal of retrorsine and became fiber-shaped aggregates similar to those in the control group after 7 days of culture (Fig. 5a,b)*.* We also found that the number of the cells in cell fibers of the experimental group did not change during the exposure to retrorsine, started to increase just after the removal of retrorsine in the culture medium, and became comparable to those in the control group after 7 days of culture (Fig. 5c). These results indicate that cell fibers where primary rat hepatocytes can proliferate could be useful for in vitro detection of drugs that inhibit hepatic regeneration and could be efficient to evaluate whether this inhibitory effect on hepatic regeneration is temporary or permanent.

### In vivo albumin secretion function of primary rat hepatocytes that have proliferated and cultured in cell fibers

**Figure 6:**
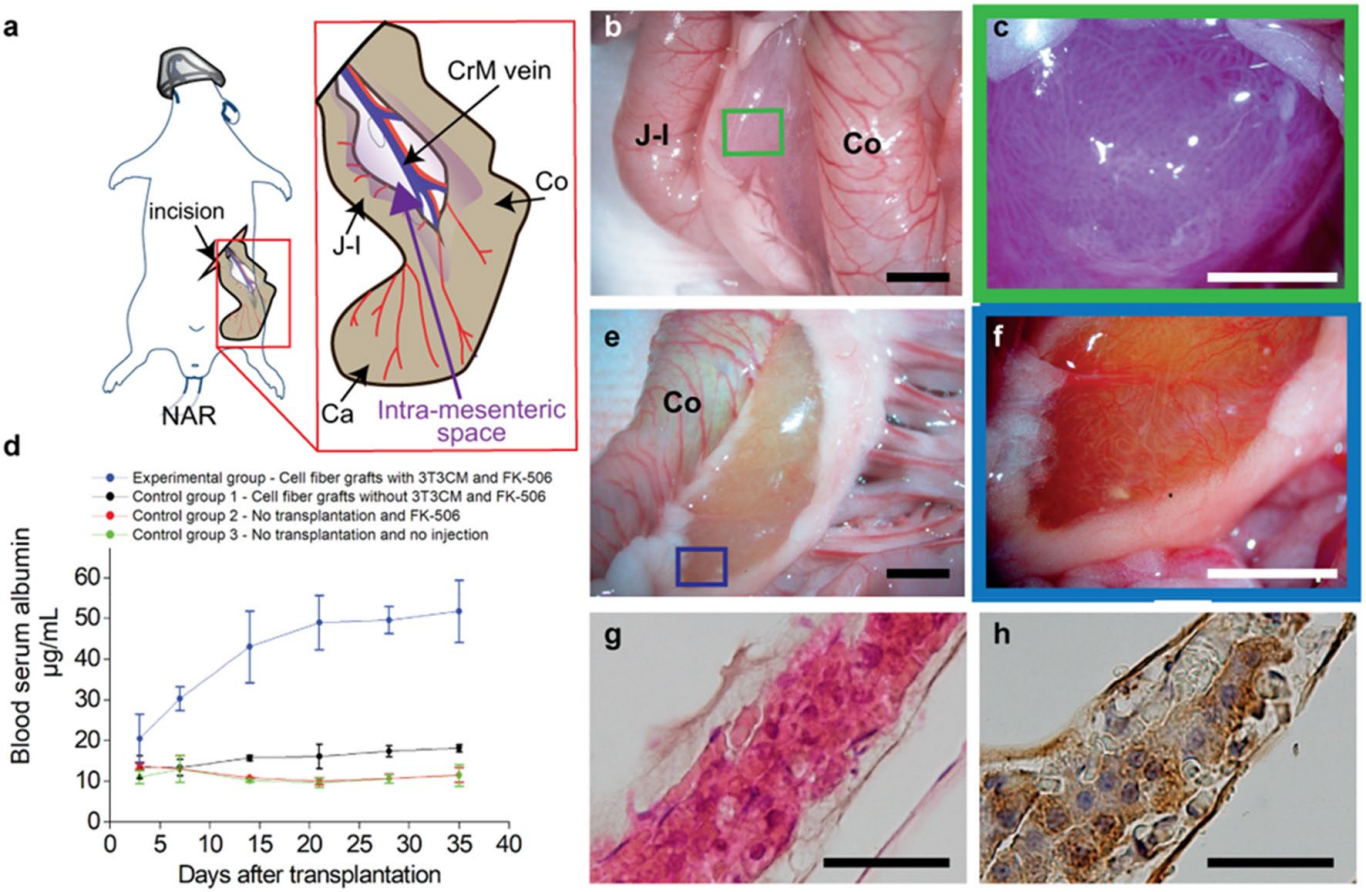
Transplantation of cell fibers encapsulating primary rat hepatocytes to analbumenic rats. (**a**) Schematic drawing of intra-mesenteric space as the transplantation site for cell fibers encapsulating primary rat hepatocytes. (**b**, **c**) Representative photo images (n = 5) with (**b**) low and (**c**) high magnifications of cell fibers in intra-mesenteric space immediately after transplantation. Scale bars; 5 mm in (**b**); 2 mm in (**c**). (**d**) Time course of plasma albumin concentrations of the analbumenic rats (NARs) in 4 groups; one group received transplants of the cell fibers cultured with 3T3 CM and daily injections of immunosuppressive drug FK-506, one group received transplants of the cell fibers cultured without 3T3 CM and the daily injections, one group received no transplants but only daily injections, one group receive neither transplants nor daily injections (n = 4 at least for each group). Error bars denote mean ± SD. *P < 0.01 and **P < 0.05; Student´s t-test at each time point. (**e**, **f**) Representative photo images (n = 4) with (**e**) low and (**f**) high magnifications of cell fibers in intra-mesenteric space 35 days after transplantation. Scale bars; 5 mm in (**e**); 2 mm in (**f**). (**g**, **h**) Representative images of histological analysis (**g**) using haematoxylin and eosin staining, and (**h**) of immunohistochemical analysis for albumin localization of primary rat hepatocytes in cell fibers retrieved 35 days after transplantation (n = 4 rats for each analysis). Scale bars; 200 µm. *CrM vein* Crania Mesenteric vein, *J–I* Jejuno–Ileum, *Ca* Caecum, *Co* Colon.

We investigated further applicability of cell fibers encapsulating primary hepatocytes to the field of medical transplantation for the treatment of a metabolic disease, namely analbuminemia^33^. For this investigation, we prepared two types of grafts and four groups of at least four Nagase analbuminemia rats (NARs). To prepare two types of grafts, core–shell hydrogel microfibers were fabricated to encapsulate primary rat hepatocytes at the initial cell seeding density of 2.5 × 10^7^ cells mL^−1^ and, 2 days after fabrication, the cell fibers were randomly divided into two groups. In one group, the cell fibers were cultured for 5 more days in media supplemented with 3T3CM resulting in proliferation of the encapsulated primary rat hepatocytes, this graft type is called "cell fiber grafts with 3T3CM". For the other graft type, cell fibers were cultured for 5 more days without being exposed to 3T3CM resulting in no proliferation of the encapsulated primary rat hepatocytes, which is called: “cell fiber grafts without 3T3CM”. Before transplantation experiments, NARs were randomly divided into four groups: one experimental group and three control groups. In the experimental group, the NARs received the cell fiber grafts with 3T3CM. In the first control group, the NARs received the cell fiber grafts without 3T3CM. For the experimental group, prior to transplantation, we evaluated the number of cells within the cell fiber grafts with 3T3CM and we transplanted the cell fibers containing approximately 2.0 × 10^7^ cells in total by putting them into each intra-mesenteric space of at least four NARs (Figs. 6a–c, S8)^34^. In parallel, for the first control group, we transplanted cell fiber grafts with the same total length as those transplanted in the experimental group, also by putting them into the same site of four NARs. All the NARs in both these groups received daily injections of tacrolimus, an immunosuppressive drug also named FK-506, after transplantation. In the second and the third control groups, the NARs received no transplant, but the former received daily injections of FK-506, and the latter received no treatment.

We found that plasma albumin concentrations of NARs increased over time up to 35 days after transplantation in the experimental group and that plasma albumin concentrations were approximately three to five times higher than those of all the control groups (Fig. 6d). Furthermore, thirty-five days after transplantation, we performed second-look analysis for NARs of the experimental group, and we observed that the transplanted cell fibers containing primary rat hepatocytes were microscopically recognizable in the intra-mesenteric spaces of all NARs (Figs. 6f, S9). In addition, histological analysis revealed that the cells in the cell fibers were morphologically intact (Fig. 6g) and that almost all these cells were stained with albumin (Fig. 6h). These results clearly show that primary rat hepatocytes that had proliferated in vitro and were cultured in cell fibers could fulfil their ability to secrete significant level of albumin in vivo, and that these cell fibers could be easily handled as transplants to treat analbuminemia. Taken together, these results confirm that the cell fibers encapsulating primary rat hepatocytes are equipped with handleable and scalable characteristics that would be essential in the context of transplantation of primary hepatocytes.

## Discussion

In the present study using cell fiber technology, we have developed a culture system offering scalability and handleability for primary rat hepatocytes that allows them both to proliferate and to retain their viability as well as metabolic functions for a long period within cell fibers, as evidenced by the observations that primary rat hepatocytes encapsulated in cell fibers increased in number under stimulation by specific environmental factors (Figs. 3 and S3) and that, subsequently, these primary hepatocytes maintained their number and viability for at least 30 days in culture while keeping their metabolic functions for the same period of time (Fig. 2). Furthermore, the evidence showing that our culture system offers capabilities of both scalability and handleability is that the primary rat hepatocytes encapsulated within cell fibers increased in number while keeping not only their sensitivity sufficient to detect the toxic effects of well-known hepatotoxic compounds such as acetaminophen and diclofenac (Table 1 and Figs. S4–S7), but also their ability to secrete albumin in adequate amounts to be observed in the blood of recipient rats with analbuminemia after being transplanted into NARs (Fig. 6). As a matter of fact, all these experimental operations were performed with stability and reproducibility certainly thanks to the ease with which the cell fibers can be fabricated, handled, and transferred.

In addition, we have shown the efficiency of our culture system to screen candidate compounds with either promoting or inhibitory effects on hepatic regeneration. Indeed, primary rat hepatocytes cultured in cell fibers under conditions stimulating proliferation did not increase in number when exposed to retrorsine, a hepatotoxic compound known to disrupt the division of rat hepatocytes; however, after ceasing exposure to retrorsine, the primary rat hepatocytes seemed to start proliferating and eventually formed fiber-shaped cellular aggregates (Fig. 5). We also believe that this capability of our culture system could extend to reproduce a phenomenon seen in vivo: in retrorsine-treated rats, liver regeneration is delayed compared to non-treated control rats after undergoing partial hepatectomy, which exerts strong proliferative stimulus on the liver by activating several pathways including raising growth factors like HGF and EGF^35–37^ but the retrorsine-treated rats eventually regenerated their liver mass completely^32^. Additionally, as a noteworthy fact showing that our culture system provides handleable cell fibers, stopping exposure to retrorsine was experimentally done by transferring the cell fibers from a medium supplemented with retrorsine to another medium without retrorsine; these procedures could probably allow the on–off switch of the effect of retrorsine to get sharpened while minimizing the damage to the primary rat hepatocytes encapsulated within cell fibers.

Moreover, in this study, we have demonstrated that our culture system can be applied to the field of medical transplantation by confirming that, after increasing in number within cell fibers, the primary rat hepatocytes improved plasma albumin concentrations in analbuminemia rats (Fig. 6). We believe that cell fibers encapsulating primary hepatocytes possess two advantageous key aspects in this field. As a qualitative aspect, the primary hepatocytes encapsulated in cell fibers connect with each other through ECM proteins, and are expected to fulfil better hepatocyte-specific functions over a longer period of time than the ones exhibited by the same number of dispersed primary hepatocytes^34^. The superiority of cell fibers could also be seen when compared with hydrogel microcapsules embedding hepatocytes lacking ECM proteins that are used in a clinical study to treat acute liver failure in children^38^ as well as in a preclinical study to treat a model of fulminant liver failure in baboons^39^. Furthermore, as a quantitative aspect, our cell fibers allow primary hepatocytes to proliferate and can expand the supply of primary hepatocytes, potentially resulting in improvement in transplant preparation especially when isolated primary hepatocytes are in short supply.

Taken together, here we have shown that cell fibers could serve to establish a culture system offering scalability and handleability for primary rat hepatocytes that are cultured under biomimetic microenvironment for long periods; within the cell fibers, primary rat hepatocytes have proliferated and subsequently maintained their viability as well as their specific hepatic functions for up to 30 days. Adding on to these features, scalability and handleability of cell fibers have demonstrated stability and reproducibility required in drug screening and medical transplantation. We believe that these features of cell fibers should lead to innovative strategies and promising developments in applying primary hepatocytes to both pharmaceutical and medical fields.

Finally, regarding the species origin of the primary hepatocytes, in the present study, we used laboratory rats under the consideration that they would be preferred for the initial development stage of our culture system for primary hepatocytes, because laboratory small animals could supply primary hepatocytes possessing a stable quality through a whole series of experiments, and rats can provide a sufficient quantity of primary hepatocytes for each experiment of this stage. As the next step toward establishment of our culture system for primary hepatocytes, we are planning to use primary human hepatocytes. Although there is an interspecies difference in the function of primary hepatocytes between rats and human^40^, we are quite optimistic about the development of such a culture system using human primary hepatocytes, since Rose et al. has paved the way for this kind of development using spheroids^12^, and we also have already succeeded in developing cell fibers from different types of human-derived cells^23–25,41^.

## Material and methods

### Hepatocyte isolation

All animal experiments in this study were approved in advance by the Ethics Committee for Animal Experiments at the University of Tokyo (approved protocol ID: 26–12, 27–18) and were conducted in compliance with the Guidelines for Research with Experimental Animals of the University of Tokyo and the ARRIVE guidelines. Primary hepatocytes were isolated from twenty-two male Sprague–Dawley rats, aged 6–9 weeks (Japan SLC, Shizuoka, Japan) as previously described^42^. Isolated rat hepatocytes with a viability of more than 85% as determined by trypan blue exclusion test were used.

### Cell fiber formation to encapsulate primary rat hepatocytes

The cell fibers were formed by using the double-coaxial laminar-flow microfluidic device assembled from pulled glass capillary tubes (inner diameter: 0.6 mm; pulled tip diameter: $\sim$ 230 µm; outer diameter: 1 mm, G-1, Narishige, Tokyo, Japan), rectangular glass tubes and connectors as previously reported^16^. To form the core–shell microfibers to encapsulate primary rat hepatocytes, three types of solutions were prepared: (1) primary rat hepatocytes-containing pre-gel solution of a mixture of two types of collagen, bovine type I collagen (AteloCell, IAC-50, Native collagen, KOKEN, Japan) supplemented with 10% of Matrigel (Corning, Tokyo, Japan) as ECM for the core; (2) pre-gel solution of 1.0% Na-alginate (from 80 to 120 cP; Wako Pure Chemical Industries, Japan) in saline for the shell; (3) mixture of 100 mM CaCl_2_ and 3% *w/w* sucrose solution for the sheath stream. To form the core–shell hydrogel microfibers with total diameters of 154 ± 28 µm and core diameters of 77 ± 12 µm, the flow rates of the streams in the three layers were corresponded and adjusted as follows: the flow rate of the stream in the core, the shell, the sheath was 20 µL min^−1^, 80 µL min^−1^, and 3.6 mL min^−1^, respectively.

### Cell counting and viability assay

Counting primary rat hepatocytes and evaluating their viability per cell fiber were performed three times and averaged. Prior to those procedures, the alginate shells were removed, and the primary rat hepatocytes were retrieved. To remove the shells of the cell fibers, 4 mg mL^−1^ of alginate lyase (Sigma Aldrich) in Dulbecco's Phosphate-Buffered Saline (DPBS) (+) was added at a 1:100 ratio to the culture media, and the media were incubated for 15 min. To retrieve primary rat hepatocytes from ECM, 4 mg mL^−1^ of collagenase (Sigma-Aldrich) in DPBS (+) was added at a 1:50 ratio to the culture media and the media were incubated for 5 min. The cell number of primary rat hepatocytes per cell fiber was counted by using cell-counting plate (WakenBtech, Japan). The cellular viabilities were examined by the trypan blue dye-exclusion test.

### Measurement of albumin and urea levels

The amount of albumin in culture medium was measured using an enzyme-linked immunosorbent assay kit (rat albumin ELISA kit, Bethyl Laboratories, Montgomery, TX). The amount of urea in culture medium was measured using an assay kit (QuantiChrom urea assay kit, BioAssay Systems, Hayward, CA). For both assays, respective absorbency at 450 nm or at 430 nm was measured using a microplate reader (MTP-810, Corona Electric, and Hitachi, Japan). All the values were normalized by the number of hepatocytes and the data were expressed as protein amount 10^–6^ cells time^−1^.

### CYP assay

Activity of cytochrome P450 1A1 enzyme was assessed using ethoxyresorufin-O-deethylase (EROD) assay as described previously^32^. Briefly, 48 h before EROD assay, primary rat hepatocytes cultured either in cell fibers or in collagen-coated well-plates started to be treated with 3 mM of the CYP1A1 inducer, 3 methylcholanthrene (3-MC; Sigma-Aldrich), contained in DMEM with a final concentration of 0.1% of dimethylsulfoxide (DMSO; Sigma-Aldrich). The media containing inducers were changed daily during the treatment. In the procedures of EROD assay, inducer-treated primary rat hepatocytes were incubated with 25 µM of EROD substrate for 1 h and subsequently the absorbency of samples at 530 nm was measured by a microplate reader (MTP-810, Corona Electric, Japan).

### Flow cytometric analysis

Using flow cytometry, primary rat hepatocytes in cell fibers were identified by detecting Asialoglycoprotein receptor-1 (ASGPR-1) based on previous report^33^. First, the hydrogel shells of the cell fibers are dissolved using alginate lyase (Sigma Aldrich), and the cells of the core were dissociated using collagenase (Brightase, Nippi, Japan). These cells were resuspended in DMEM supplemented with 10% of FBS, incubated for at least 30 min, and then centrifuged at 400 g. The cells were treated for 30 min with a 100 µL solution containing a mouse-anti-human ASGPR1 antibody (Thermofisher, Waltham, MA) diluted 1:50. A fluorescein isothiocyanate (FITC)-labeled goat anti-mouse antibody (Thermofisher, Waltham, MA) was added to the solution at the concentration of 0.5 g mL^−1^, and the cells in the solution was incubated for 30 min. The cells were washed 3 times with 1 mL fluorescence-activated cell sorter buffer (1% sodium azide and 2% fetal bovine serum in phosphate-buffered saline), resuspended in 500 µL fluorescence-activated cell sorter buffer, and analyzed by flow cytometry. Cell analysis was performed on a Becton–Dickinson FACSVerse flow cytometer (Becton–Dickinson Biosciences, San Jose, CA) and data acquisition and analysis was performed using Becton–Dickinson FACSuite software (Becton–Dickinson Biosciences, San Jose, CA).

### Histological analysis

Cell fibers containing primary rat hepatocytes were fixed in 4% paraformaldehyde in a 0.1 M phosphate buffer at pH 7.4. All samples were immersed in the fixative individually for 6 h. The samples were dehydrated through graded series of ethanol, immersed into xylene, and embedded in paraffin according to the standard methodology. Then, 5–6 µm-thick sections were obtained. For H&E staining, the sections were de-waxed, underwent hematoxylin and eosin. For immunohistochemistry, the sections were de-waxed and were incubated in blocking buffer (1% goat serum in PBS). To detect albumin, rabbit anti-rat albumin antibody (RaRa/ALB/7S, Nordic-MUbio, Netherlands) was used as primary antibody and biotinylated goat anti-rabbit antibody (VECTASTAIN Elite ABC HRP Kit, Vector Laboratories, CA) were used as secondary antibody. To visualize the antigen–antibody reaction, the sections were incubation in a 0.05 M Tris–HCl buffer (pH 7.6) containing 0.01% 3,3′-diaminobenzidine and 0.001% H_2_O_2_. The sections were observed under a light microscope (BX 53; Olympus, Tokyo, Japan).

### Hepatotoxicity assessment

Acetaminophen (Sigma-Aldrich) and diclofenac (Sigma-Aldrich) were adopted as hepatotoxic compounds and stocked in DMSO at the concentration of 10 mM and 1 mM, respectively. Primary rat hepatocytes cultured either in cell fibers or in collagen-coated 24-well plates were treated for 24 h with either of these compounds diluted in media at intended concentrations, and then the respective compound-treated hepatocytes underwent assays of their different specific characteristics: cell viability, albumin secretion, or urea synthesis. Data from drug-treated hepatocytes were normalized to the respective untreated controls in the same culture system. By plotting these normalized data on y-axis and the concentrations of compounds on x-axis, the concentration-reaction curves were generated. Afterwards, using Origin software (Origin 2020, Origin Lab, Northampton, MA), IC50 values were estimated as the interpolated concentrations at which 50% of the hepatocytes are supposed to have lost their characteristics.

### Assessment of hepatocyte proliferation inhibition

Retrorsine (Sigma-Aldrich) was adopted as a compound that inhibits hepatocyte proliferation. The working solution of retrorsine was prepared through the following two steps: (1) retrorsine was added into distilled water at 20 mg ml^−1^ and dissolved completely through titration of the solution to pH 2.5 using 1 N HCl; (2) this solution was neutralized using 1 N NaOH, and NaCl was added into this solution at 150 mM. Then immediately, this working solution was diluted in media at intended concentrations. The changeover between starting and stopping exposure to retrorsine was done by medium replacement; the culture media surrounding the cell fibers can be completely removed from petri dishes to be replaced with new ones while keeping the cell fibers intact. The proliferation rate of the hepatocytes in the experimental groups was calculated by dividing the numbers of the hepatocytes that proliferated from 2 days of culture to 3 days of culture in the experimental groups with the ones in the control group. The concentration-reaction curves were generated by plotting these proliferation rates on the y-axis and the concentrations of retrorsine on x-axis. Thereafter, using Origin software (Origin 2020, Origin Lab, Northampton, MA), IC50 value for retrorsine was then estimated as the interpolated concentrations at which 50% of the hepatocytes are supposed to have been inhibited in proliferation.

### Cell fiber transplantation for NARs

Six-week-old male NARs were purchased from Japan SLC (Shizuoka, Japan). The rats were conditioned for the experiments by being housed in plastic cages in a room at the controlled temperature of 23 ± 2 °C. Eighteen rats, weighing more than 240 g, underwent surgical procedures for transplantation of the cell fibers as follows: the rat was anesthetized by 2.0% isoflurane (isoflurane for Animal, Intervet, Tokyo) delivered using an animal anesthetizer device (MK-AT210D, Muromachi Kikai, Tokyo). Cell fibers encapsulating 2 × 10^7^ primary rat hepatocytes that had been cultured for 7 days via their proliferation under 3T3CM were rinsed with serum-free culture medium once. The cell fibers were then sucked into a 23 G butterfly needle (SURFLO, Terumo, Tokyo) connected with a 20 mL syringe. After laparotomy of the rat, small and large intestines were exposed, and the cell fibers were injected into intramesenteric space and placed along the portal vein. The rat received subcutaneous injection of tacrolimus, FK-506 (Prograf, Astellas Pharma, Tokyo) at 1 mg kg^−1^ body weight every day after transplantation. Thirty-five days after transplantation, the rat underwent second-look laparotomy under the same anesthesia used for the transplantation procedure, and the transplanted cell fibers encapsulating primary rat hepatocytes were removed. 350 μL whole blood were sampled from the rat through its tail vein before and every 7 days up to 35 days after transplantation, and the plasma albumin concentrations were measured using ELISA assay (Rat Albumin ELISA Quantitation Set, Bethyl Laboratories, TX).

### Statistical analysis

Data were processed with Origin 2020 (Origin Lab, Northampton, MA). Each experiment was carried out with at least three individual samples. All the values in results were given as the mean ± standard deviation (SD). Statistical analysis was performed by using Student’s t-test, with *P* = 0.05 or P = 0.01 considered to be statistically significant, if not stated differently. For in vitro detection of drug hepatotoxicity (Table S3), we additionally assessed for the significance of the differences between groups by using one-way analysis of variance (ANOVA).

## Supplementary Information

Supplementary Information.
