# Supplementary material for: In vitro proliferation and long-term preservation of functional primary rat hepatocytes in cell fibers
Source: Sci Rep. 2022 May 25;12:8813. doi: 10.1038/s41598-022-12679-3 (PMC9133069; doi:10.1038/s41598-022-12679-3)
Supplement: Supplementary file 1 — Supplementary Information. [file 41598_2022_12679_MOESM1_ESM.pdf]

In Vitro Proliferation and Long-Term Preservation of Functional Primary Rat Hepatocytes in Cell Fibers

Supplementary information

Elsa Mazari-Arrighi[a,b,c,e,f], Teru Okitsu[a,b*], Hiroki Teramae[d], Hoshimi Aoyagi[a,b], Mahiro Kiyosawa[a,b], Mariko Yano[a,b], François Chatelain [e,g], Alexandra Fuchs [e,g], Shoji Takeuchi[a,b*]

[a] Institute of Industrial Science, The University of Tokyo, Japan.

[b] ERATO Takeuchi Biohybrid Innovation Project, Japan Science and Technology Agency (JST), Japan.

[c] LIMMS/CNRS-IIS UMI 2820, Institute of Industrial Science, The University of Tokyo, Japan

[d] Faculty of Teacher Education, Shumei University, Chiba, Japan.

[e] Université de Paris, Inserm, U976 HIPI, F-75006, Paris, France.

[f] AP-HP, Hôpital Saint-Louis, Paris, France.

[g] IRIG/DRF, CEA, F-38000, Grenoble, France

Table of contents

1. **Supplemental methods**
2. **Supplemental figures and tables**

1. Supplemental methods

Culture of primary rat hepatocytes in cell fibers

After formation of core-shell hydrogel microfibers encapsulating primary rat hepatocytes, they were incubated at 37 ºC for more than 15 minutes to set the ECM proteins at the core region into gels. Subsequently, primary rat hepatocytes in cell fibers were cultured in DMEM supplemented with 1% Glutamax, 10% heat-inactivated FBS, 100 IU mL^{-1} penicillin, 100 mg mL^{-1} streptomycin, insulin, 6.25 mg mL^{-1} transferrin, 6.25 ng mL^{-1} selenous acid (Lonza, Tokyo, Japan), and 1mM HEPES at 37 ºC under a water saturated 5% CO_2 environment; this medium is the one that have been optimized to culture primary rat hepatocytes in cell fibers for long term (**Figure S1c**). Media exchanges were performed every 48 hours.

When primary rat hepatocytes were cultured either using cell fibers or using collagen-coated 24-well plates, the morphologies of these primary cells were observed using an inverted fluorescence microscope (IX71; Olympus, Tokyo, Japan).

Culture of primary rat hepatocytes using collagen-coated 24-well plates

$5x10^5$ primary rat hepatocytes freshly isolated were seeded on collagen-coated 24-well plates (Corning Japan K.K, Tokyo, Japan). The primary rat hepatocytes were first cultured in Dulbecco's Modified Eagle's Medium (DMEM; Gibco, Tokyo, Japan) supplemented with 1% Glutamax (Life Technologies, Tokyo, Japan), 10% heat-inactivated foetal bovine serum (Equitech-Bio, Kerrville, TX), 100 IU mL^{-1} penicillin (Gibco), 100 mg mL^{-1} streptomycin (Gibco), and 1mM HEPES (Sigma Aldrich, Japan) at 37 ºC under a water saturated 5% CO_2 environment. Three hours after the beginning of culture, media were changed and the primary rat hepatocytes started to be cultured in William's medium E (Life Technologies) supplemented with 1% Glutamax, 100 IU mL^{-1} penicillin, 100 mg mL^{-1} streptomycin, 6.25 mg mL^{-1} insulin, 6.25 mg mL^{-1} transferrin, 6.25 ng mL^{-1} selenous acid (Lonza, Tokyo, Japan), 10 mM HEPES, 40 ng mL^{-1} dexamethasone (Sigma-Aldrich), and 2% of Matrigel (Corning). Media exchanges were performed every 24 hours.

Cell fiber formation to encapsulate and culture NIH/3T3 fibroblasts

Mouse embryonic fibroblast cell line, NIH/3T3 cells (TKG0299, RIKEN Cell Bank, Japan) were maintained using petri dishes containing DMEM with 1% Glutamax, 10% FBS (Thermo Scientific, Waltham, MA), 100 IU mL^{-1} penicillin, and 100 mg mL^{-1} streptomycin. The cells were cultured in a humidified incubator at 37 ºC with 5% CO_2.

When they reached confluence, the cells were harvested by trypsin/EDTA (Gibco) treatment and were encapsulated into core-shell hydrogel microfibers using the same protocol as used for encapsulation of primary rat hepatocytes except for the condition regarding ECM proteins for the core solution: bovine type I collagen (AteloCell, IAC-50, Native collagen, KOKEN, Japan) alone.

Preparation of 3T3CM to stimulate primary rat hepatocytes for proliferation in vitro

3T3CM was used as the media supplement for primary rat hepatocytes to proliferate according to previous report.[31] The duration and the conditions of culturing NIH/3T3 cells before their encapsulation into cell fibers were determined based upon the findings in our preliminary experiments. Specifically, the amount of HGF in the conditioned medium is always higher when NIH/3T3 cells are cultured in cell fibers than when cultured in petri dishes (**Figure S2**), and the HGF amount is highest after 5 days of culture when cells are cultured in cell fibers. Furthermore, the 3T3CM collected from in cell fibers has showed comparable effects on maintenance of both microscopic morphology of fiber-shaped hepatic cell aggregates as well as hepatocyte-specific functions, namely albumin secretion and urea synthesis, with the media supplemented with both recombinant mouse HGF and recombinant mouse EGF (**Figure S3**). Based on the findings as described above, we decided to use the culture media supplemented with 3T3CM that had been cultured in cells fibers for 5 days, and in this study, to make primary rat hepatocytes to proliferate, the media supplemented with 3T3CM started to be used after 2 days of culture. Since the start of using 3T3CM, culture media were exchanged every 24 hours.

2. Supplemental figures and tables

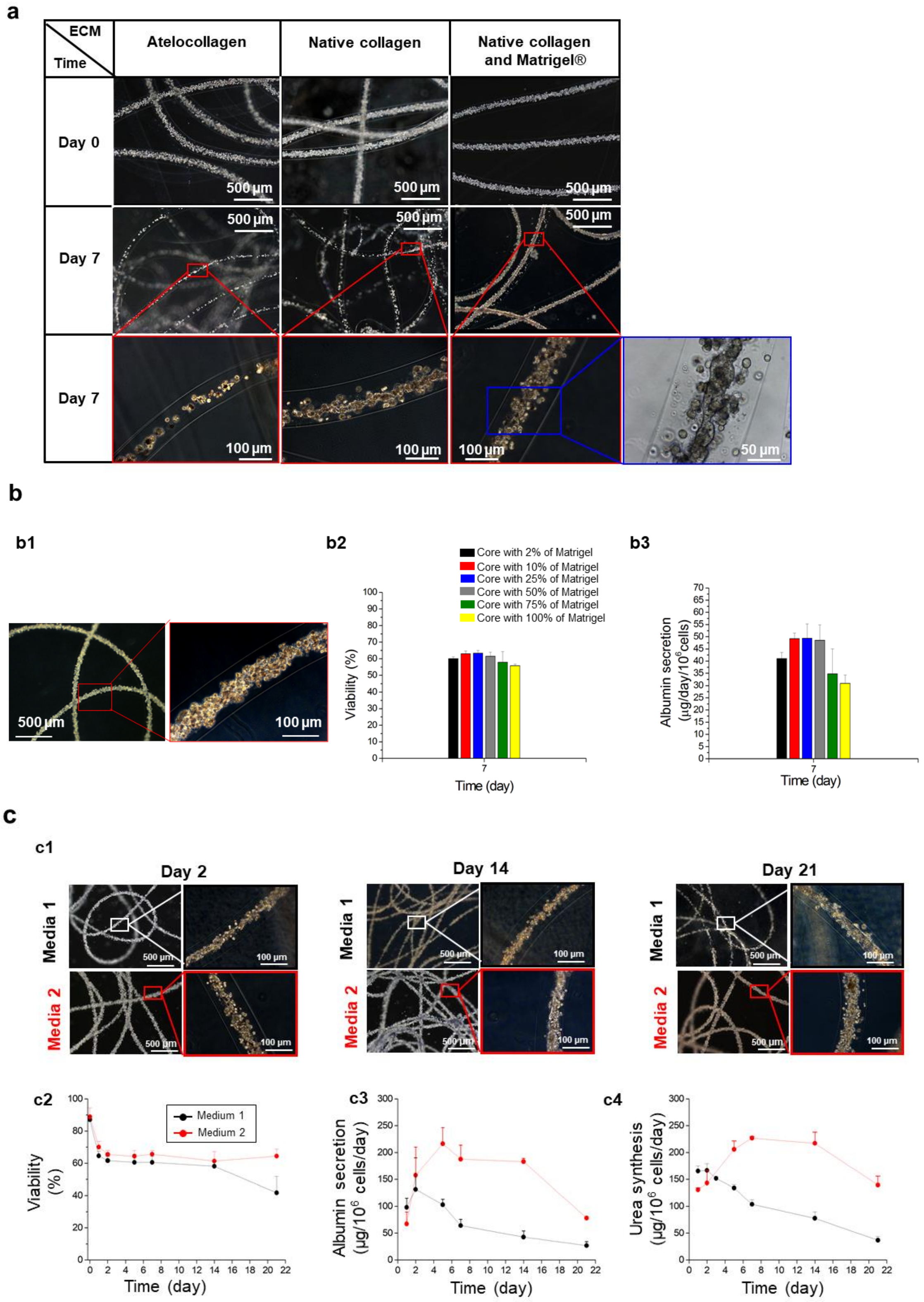

Figure S1. Preliminary experiments to select key parameters for encapsulation of primary hepatocytes into cell fibers.

(a) Influence of ECM protein composition in the core of cell fibers on the behaviours of primary rat hepatocytes encapsulated within cell fibers at the initial cell seeding density of 9.10^7 cells/ml and cultured for 7 days. **(b)** Influence of Matrigel concentration in the core of cell fibers on the primary rat hepatocytes encapsulated within cell fibers at the initial cell seeding density of 9.10^7 cells/mL and cultured for 7 days. **(b1)** Representative dark-field images of primary rat hepatocytes encapsulated in cell fibers with native collagen as well as with 10% of Matrigel and cultured for 7 days. **(b2-b3)** Impact of Matrigel concentration in the core composed of primary rat hepatocytes on **(b2)** viability examined by the blue Trypan exclusion test and on **(b3)** albumin secretions in the culture medium, which was measured by ELISA assay. (c) Influence of 3D culture medium formulation on the behaviours of primary rat hepatocytes encapsulated within cell fibers at the initial cell seeding density of 9.10^7 cells/ml and cultured over a 3-week period. **(c1)** Representative dark-field images of rat primary hepatocytes within cell fibers cultured in medium 1* and in medium 2** at day 2, day 4, and day 21. **(c2)** Monitoring of hepatocyte viability. **(c3)** Time-course changes of albumin secretion. **(c4)** Time-course change of urea synthesis in the culture medium. N≥4 per condition. Error bars denote mean ± SD.

*Medium 1 = William's medium supplemented with 1% Glutamax, 100 IU mL^{-1} penicillin, 100 mg mL^{-1} streptomycin, 6.25 mg mL^{-1} insulin, 6.25 mg mL^{-1} transferrin, 6.25 ng mL^{-1} selenous acid, 10 mM HEPES, 40 ng mL^{-1} dexamethasone, and 2% of Matrigel
**Medium 2 = DMEM supplemented with 1% Glutamax, 10% heat-inactivated FBS, 100 IU mL^{-1} penicillin, 100 mg mL^{-1} streptomycin, 6.25 mg mL^{-1} insulin, 6.25 mg mL^{-1} transferrin, 6.25 ng mL^{-1} selenous acid, and 1mM HEPES.

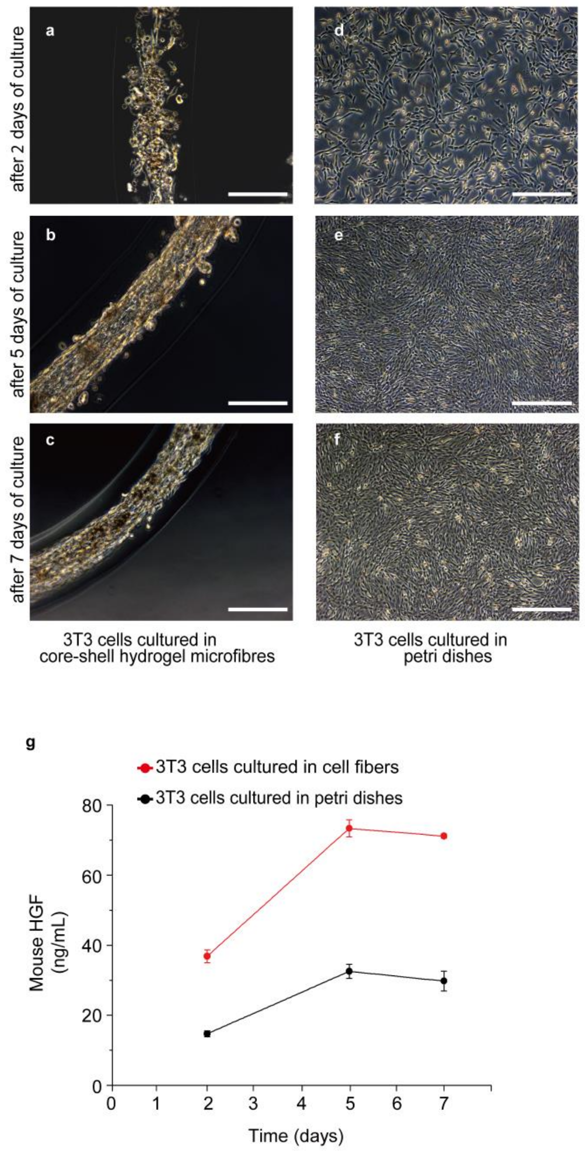

Figure S2. Culture of NIH/3T3 cells either in cell fibers or in collagen-coated petri dishes to prepare NIH/3T3 cell conditioned medium.
(**a-c**) Representative dark-field images (n=4 cell fibers) of NIH/3T3 cells encapsulated in cell fibers after 2 days, 5 days and 7 days of culture. Scale bars; 75 µm. (**d-e**) Representative dark-field images (n=4 petri-dishes) of rat hepatocytes in collagen-coated petri-dishes after 2 days, 5 days and 7 days of culture. Scale bars; 75 µm. (**g**) The concentrations of mouse hepatocyte growth factor secreted by NIH/3T3 cells cultured either in cell fibers and in collagen-coated petri dishes over 7 days of culture (n=4 per data point).

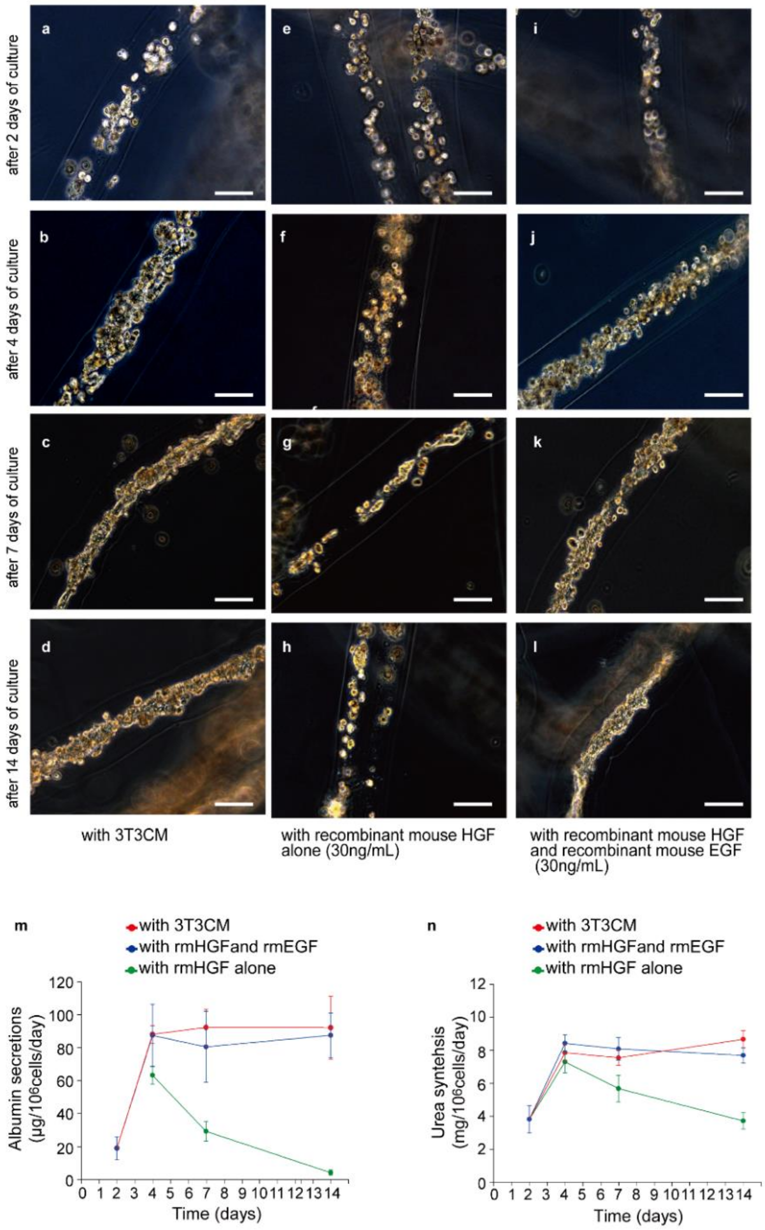

Figure S3. Culture of primary rat hepatocytes in cell fibers for up to 14 days under various proliferation stimulations.

(**a-l**) Representative dark-field images (n=6 cell fibers) of rat hepatocytes encapsulated in cell fibers at the initial cell density of 2.5x10^7 cells mL^{-1} after 2, 4, 7, 14 days of culture either with 3T3CM, with recombinant mouse hepatocyte growth factor (HGF) alone (rmHGF alone), or with recombinant mouse HGF and recombinant mouse epidermal growth factor (EGF) (rmHGF and rmEGF). Scale bars; 75 µm. (**m-n**) Concentrations of albumin secreted (**m**) and urea synthesized (**n**) by primary rat hepatocytes cultured in cell fibers (n=3 at least per data point) over 14 days either with 3T3CM, with rmHGF alone, or with rmHGF and rmEGF.

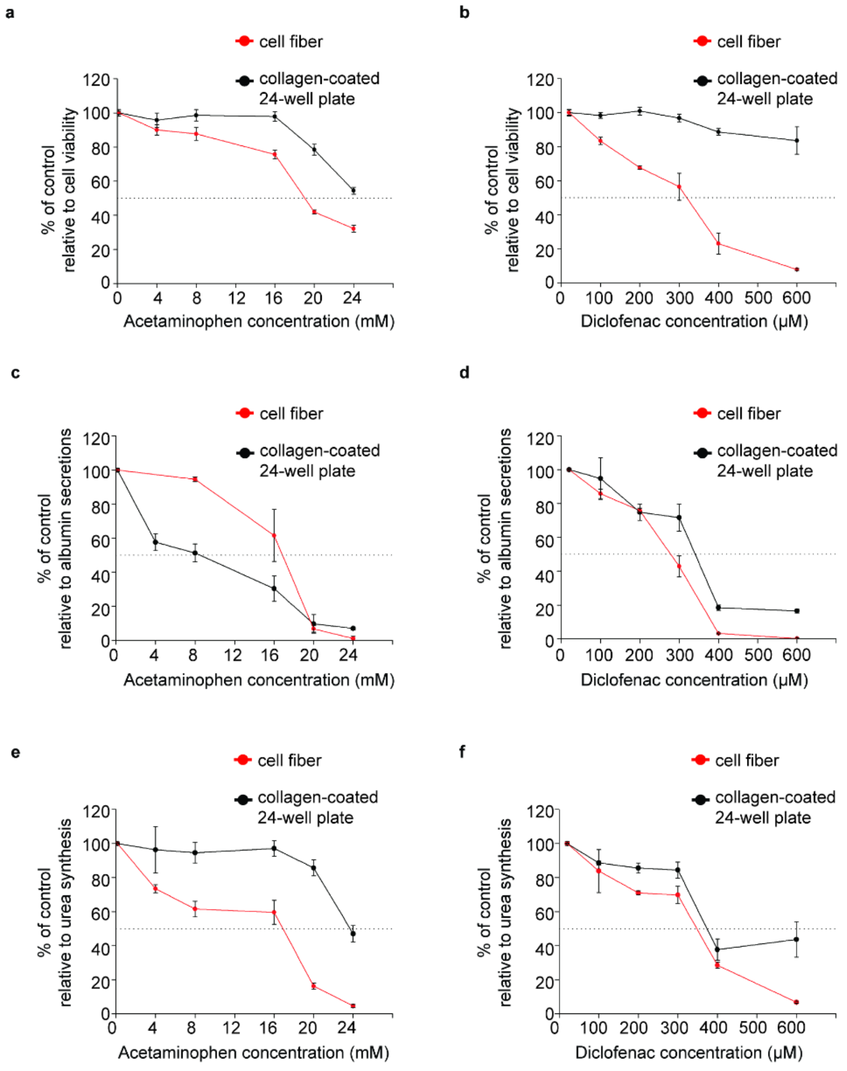

Figure S4. Concentration-reaction curves for acetaminophen and diclofenac relating to viability, albumin secretion, and urea synthesis of primary rat hepatocytes cultured either in cell fibers or in collagen-coated petri dishes after 4 days of culture.

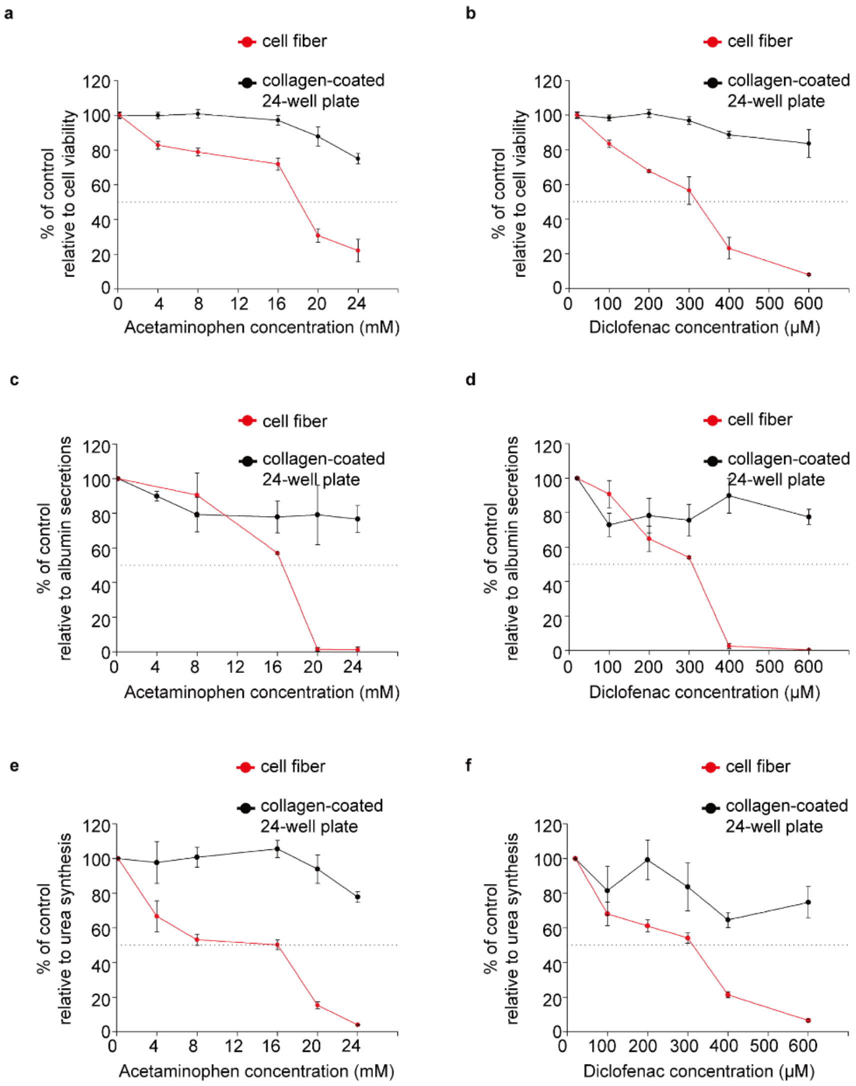

Figure S5. Concentration-reaction curves for acetaminophen and diclofenac relating to viability, albumin secretion, and urea synthesis of primary rat hepatocytes cultured either in cell fibers or in collagen-coated petri dishes after 7 days of culture.

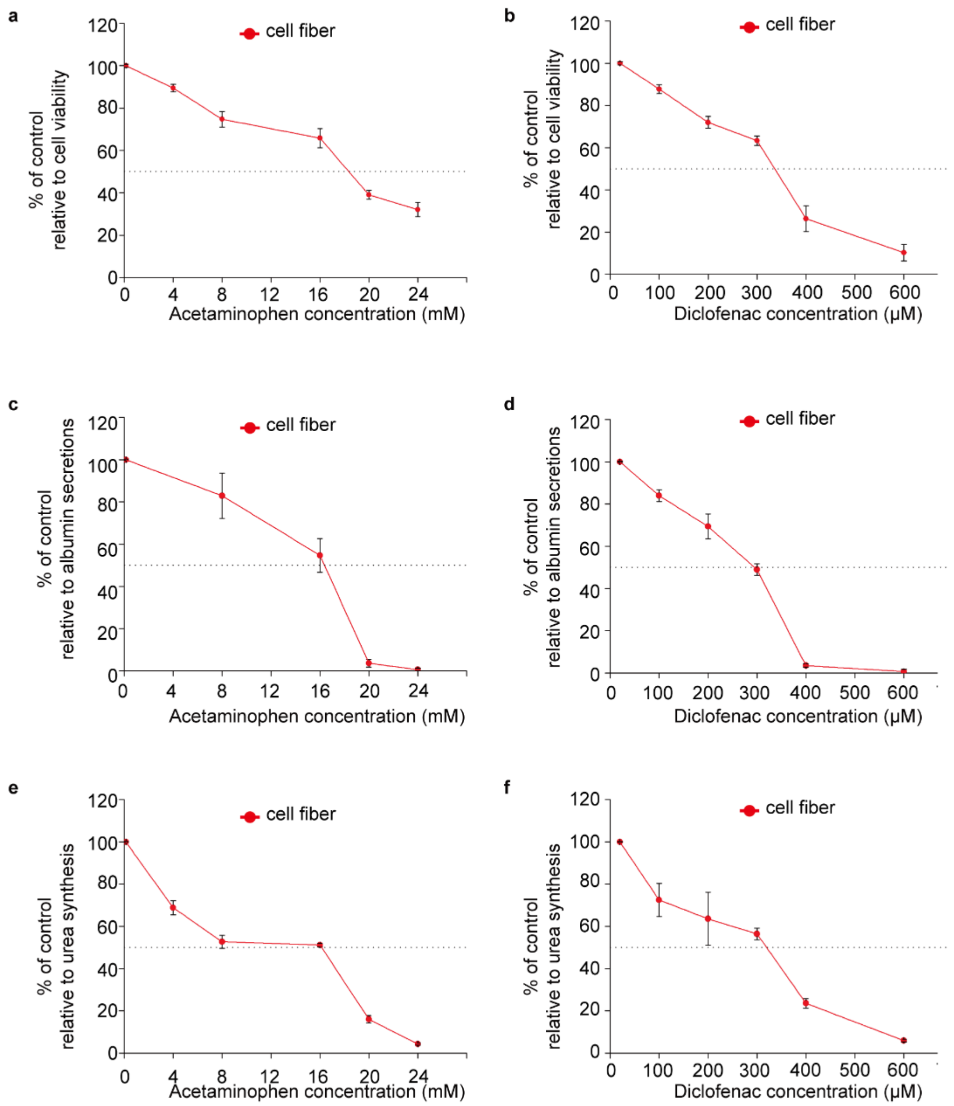

Figure S6. Concentration-reaction curves for acetaminophen and diclofenac relating to viability, albumin secretion, and urea synthesis of primary rat hepatocytes cultured in cell fibers after 14 days of culture.

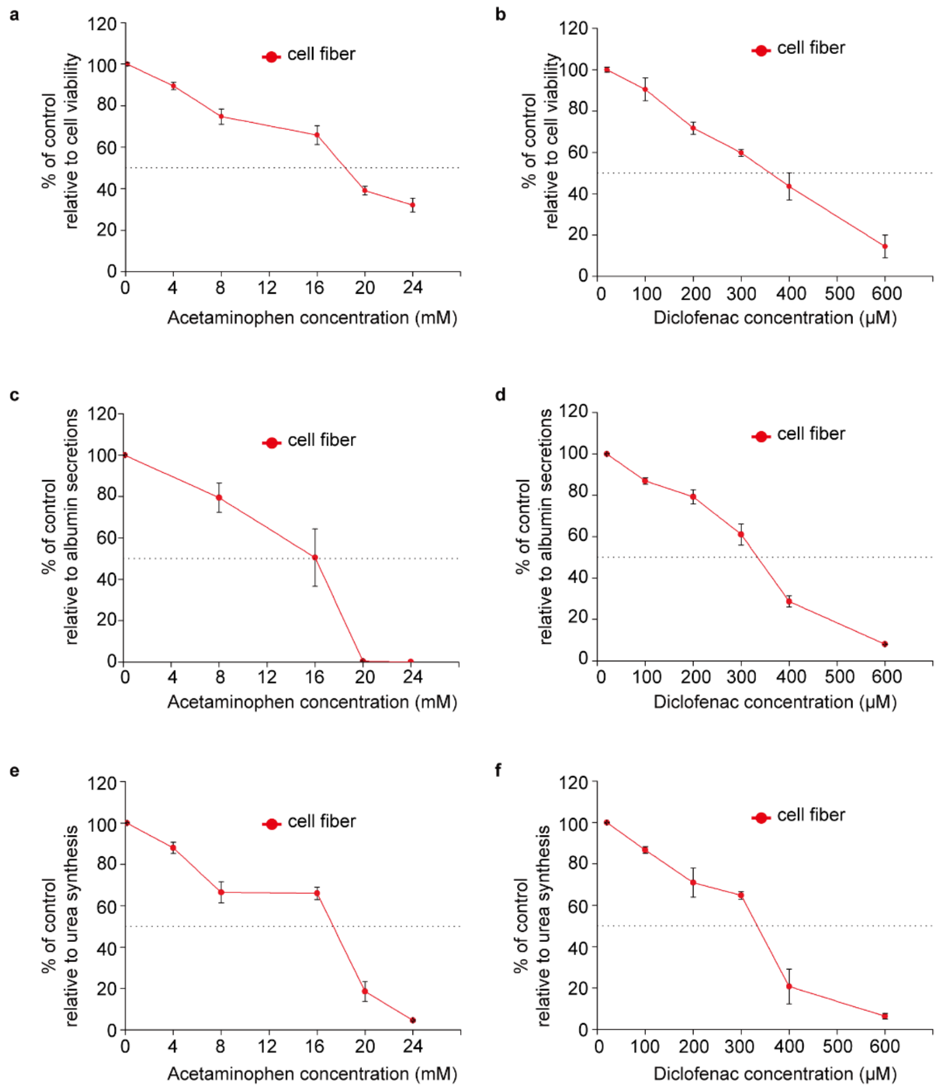

Figure S7. Concentration-reaction curves for acetaminophen and diclofenac relating to viability, albumin secretion, and urea synthesis of primary rat hepatocytes cultured in cell fibers after 30 days of culture.

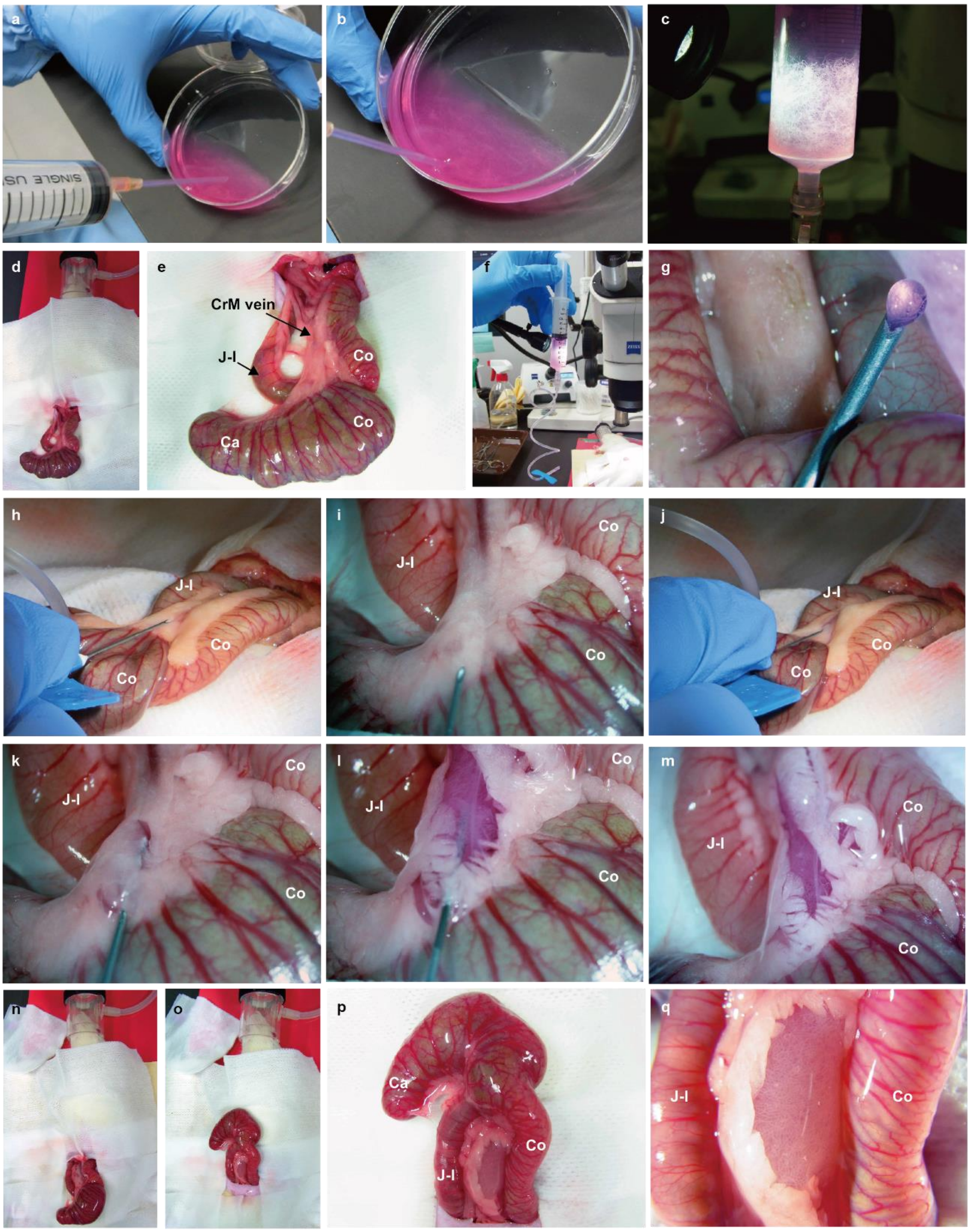

Figure S8. Transplantation of cell fibers encapsulating primary rat hepatocytes into the intra-mesenteric space of an analbumenic rat.
(**a, b**) Cell fibers encapsulating primary rat hepatocytes were rinsed with serum-free culture medium once. (**c, f**) The cell fibers were sucked into a 23G butterfly needle connected with a 20 mL syringe. After laparotomy of the rat, (**d, e**) small and large intestines were exposed, and (**g-q**) the cell fibers were injected into intramesenteric space and placed along the portal vain. CrM vein, Crania Mesenteric vein; J-I, Jejuno-Ileum; Ca, Caecum; Co, Colon.

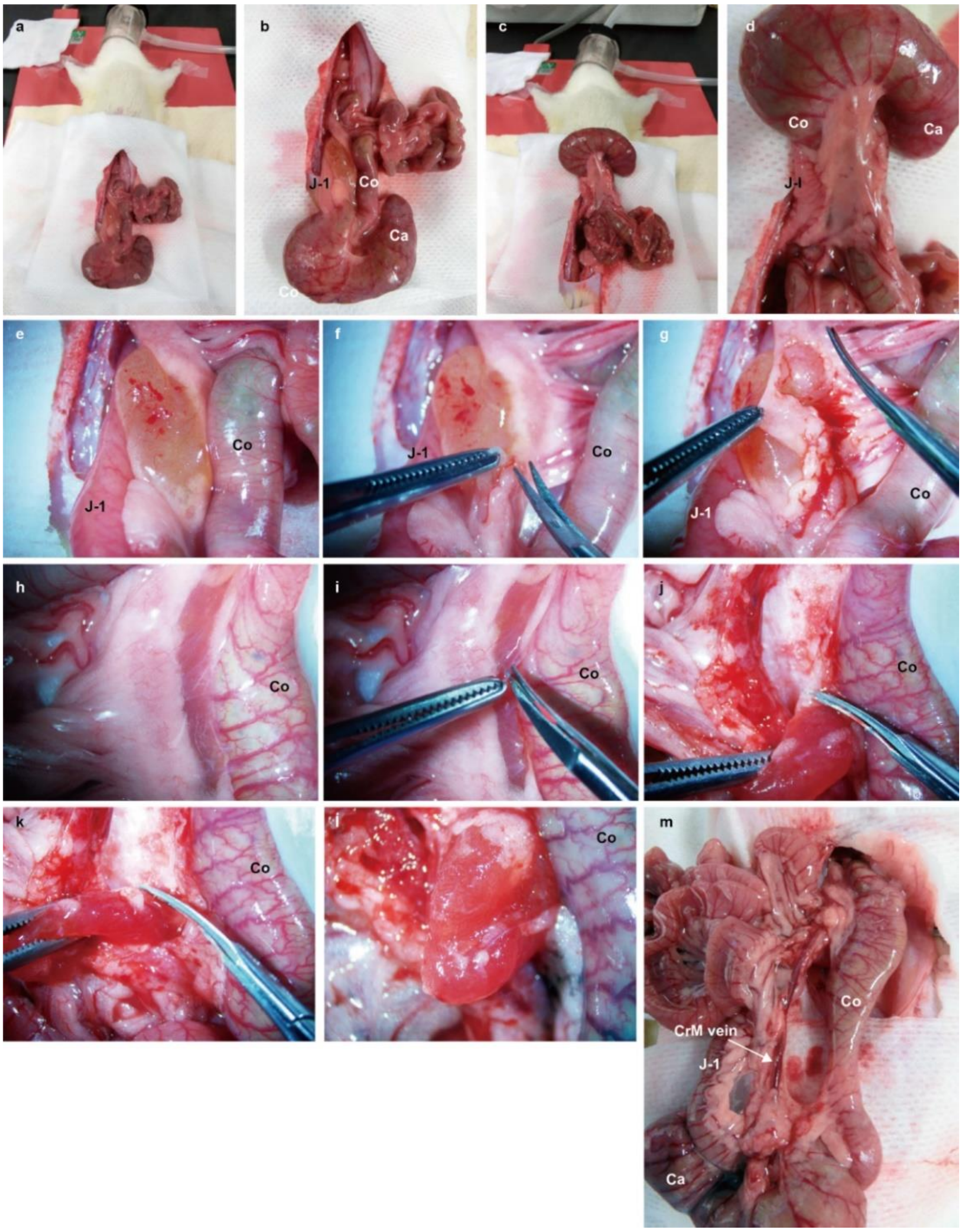

Figure S9. Resection of cell fibers encapsulating primary rat hepatocytes transplanted into the intra-mesenteric space of an analbumenic rat 35 days after transplantation.

(**a-e**) Thirty-five days after transplantation, the rat underwent second-look laparotomy under anesthesia, and (**f-m**) the transplanted cell fibers encapsulating primary rat hepatocytes were removed. CrM vein, Crania Mesenteric vein; J-I, Jejuno-Ileum; Ca, Caecum; Co, Colon.

Table S1. Proportion of hepatocytes in primary rat hepatic cells cultured in cell fibers using 3T3CM, before culture, after 7, 30, and 90 days of culture.

Culture duration (days)	Total hepatic cell number ($x10^5$ cells)	Average proportion of ASGPR1-positive cells (%)	ASGPR1-positive cell number ($x10^5$ cells)	Number of samples
Before culture	2.34±0.20	89.89±5.04	2.19±0.15	4
7	6.71±0.46	71.82±11.25	4.81±0.75	3
30	3.86±0.13	57.38±6.03	2.27±0.17	3
90	0.84±0.08	51.09±3.86	0.43±0.07	3

Table S2. 50% inhibitory effect (IC50 values) for acetaminophen and diclofenac relating to viability, albumin secretion, and urea synthesis of primary rat hepatocytes cultured either in cell fibers for 4 days, 7 days, 14 days, and 30 days, or in collagen-coated 24-well plates for 4 days and 7 days

Data are presented as the mean ± standard deviation of at least four independent cell fibers and four collagen-coated wells from collagen-coated 24-well plates.

**Compound**	**Culture system**	**Culture duration (days)**	**IC50 value (μM)**		
			Assay methods		
			Viability	Albumin secretion	Urea synthesis
Acetaminophen	Cell fibers	4	18324±509	16703±2540	17034±1045
		7	18556±661	16406±992	15886±582
		14	18919±772	16273±1289	15943±490
		30	18853±661	15876±2063	17299±853
	Collagen-coated 24-well plates	4	19277±164	8507±89	24038±57
		7	N/A	N/A	N/A
Diclofenac	Cell fibers	4	332±17	282±9	350±12
		7	312±21	310±3	315±9
		14	339±12	299±5	322±7
		30	345±25	337±13	336±16
	Collagen-coated 24-well plates	4	472±98	N/A	385±41
		7	N/A	N/A	N/A

Table S3. **50% inhibitory effect (IC50 values) for acetaminophen and diclofenac relating to viability, albumin secretion, and urea synthesis of primary rat hepatocytes cultured either in cell fibers for 4 days, or in other types of systems reported previously.**

Data are presented as the mean ± standard deviation of at least four independent core-shell hydrogel microfibers in this study.

compound	culture system	culture duration	assay methods	IC50 value (μM)	source of data
acetaminophen	Cell fibers	4 days of culture	Viability	18324±509	This study
			Albumin secretion	16703±2540	
			Urea synthesis	17034±1045	
	collagen-coated plates	3 hour after plating	Viability	14014±3204	Wang *et al.*[27]
	collagen-coated plates	2 days after plating	Viability	14300±NA	Luo *et al.*[28]
	3D culture in beads	3 hour after plating	LDH	10241±N/A	Biagini *et al.*[29]
	3D culture in microfluidic device	2 days of culture	Viability	16900±N/A	Toh *et al.*[30]
diclofenac	Cell fibers	4 days of culture	Viability	332±17	This study
			Albumin secretion	282±9	
			Urea synthesis	350±12	
	collagen-coated plates	3 hour after plating	Viability	263±43.1	Wang *et al.*[27]
	collagen-coated plates	2 days after plating	Viability	139±NA	Luo *et al.*[28]
	3D culture in beads	3 hour after plating	LDH	709±62	Biagini *et al.*[29]
	3D culture in microfluidic device	2 days of culture	Viability	310±N/A	Toh *et al.*[30]

Table S4. Inhibition rates of acetaminophen and diclofenac relating to viability, albumin secretion, and urea synthesis at their various concentrations using primary rat hepatocytes in cell fibers after 4, 7, 14, and 30 days of culture.

P-value of each concentration of acetaminophen or diclofenac was assessed by using one-way analysis of variance (ANOVA). *Respective inhibition rates of viability, albumin secretion, or urea synthesis at the considered concentration point were significantly different from each other after 4, 7, 14, and 30 days of culture at the 0.05 level.

Compound	Assay method	Concentration (µM)	4 days of culture	7 days of culture	14 days of culture	30 days of culture	P-value
			**Viability inhibition ± SD (%)**				
Acetaminophen	Viability	4000	89.9±3.0	84.2±2.2	89.4±1.8	93.2±2.3	P=0.74481
		8000	87.8±3.7	78.8±2.1	74.8±3.7	84.2±2.1	P=0.00151*
		16000	74.0±2.45	69.9±3.5	65.8±4.5	68.1±6.2	P=0.12448
		20000	41.7±6.8	43.3±3.9	42.6±4.7	50.1±6.5	P=0.72592
		24000	23.9±3.9	24.3±3.0	24.6±2.1	25.6±1.8	P=0.98843
			**Albumin secretion inhibition ± SD (%)**				
	Albumin secretion	4000	90.1±1.8	89.8±2.0	91.6±1.9	88.6±2.2	P=0.69603
		8000	83.0±1.3	79.0±10.6	83.0±10.8	85.3±7.1	P=0.23401
		16000	61.5±15.2	57.0±1.6	54.7±4.0	50.5±8.9	P=0.67973
		20000	6.7±1.9	5.7±1.0	5.6±1.8	4.3±0.9	P=0.46476
		24000	1.4±1.1	1.3±1.5	1.05±0.7	0.51±0.5	P=0.26872
			**Urea synthesis inhibition ± SD (%)**				
	Urea synthesis	4000	73.4±2.4	76.6±8.9	78.8±3.3	61.5±1.1	P=0.79859
		8000	61.5±4.6	53.1±3.2	52.7±2.1	61.5±1.0	P=0.15581
		16000	59.6±7.0	50.2±2.9	51.2±0.8	66.0±3.1	P=0.00104*
		20000	16.2±1.9	15.3±2.0	16.0±1.7	18.5±4.7	P=0.45514
		24000	4.6±0.9	4.0±0.3	4.3±0.6	4.5±0.4	P=0.48949
			**Viability inhibition ± SD (%)**				
Diclofenac	Viability	100	89.5±3.3	84.4±2.1	87.8±2.1	90.4±5.5	P=0.69625
		200	74.4±1.4	69.7±2.9	72.0±2.8	71.8±3.0	P=0.61605
		300	64.5±2.8	56.7±7.9	63.3±2.3	59.8±1.6	P=0.65618
		400	22.3±7.0	23.2±6.3	26.4±6.1	38.6±6.4	P=0.17372
		600	11.5±5.0	7.9±4.1	10.3±3.9	14.4±4.4	P=0.40207
			**Albumin secretion inhibition ± SD (%)**				
	Albumin secretion	100	85.8±2.8	90.7±7.9	84.0±2.9	87.0±1.5	P=0.36030
		200	72.5±4.8	64.8±7.3	69.4±6.0	69.±3.4	P=0.79634
		300	56.3±5.3	54.0±1.7	49.0±2.8	61.0±5.2	P=0.44567
		400	3.3±0.3	2.6±1.4	3.5±0.8	28.6±2.7	P=0.00492*
		600	0.3±0.3	0.4±0.3	1.0±0.6	1.0±0.5	P=0.72488
			**Urea synthesis inhibition ± SD (%)**				
	Urea synthesis	100	81.8±10.0	68.0±6.8	72.5±7.9	69.2±6.3	P=0.07358
		200	68.5±5.9	61.1±3.4	63.6±12.5	70.9±8.0	P=0.32586
		300	59.7±5.0	54.1±2.9	56.5±2.7	62.3±3.8	P=0.11259
		400	26.0±3.5	21.3±1.7	23.6±2.2	20.7±8.4	P=0.38423
		600	6.8±0.6	6.5±0.8	5.9±0.6	6.3±0.3	P=0.56494
